# Metabolic‐Immune Suppression Mediated by the SIRT1‐CX3CL1 Axis Induces Functional Enhancement of Regulatory T Cells in Colorectal Carcinoma

**DOI:** 10.1002/advs.202404734

**Published:** 2025-01-09

**Authors:** Ruiyang Zi, Xiang Zhao, Limei Liu, Yijie Wang, Rui Zhang, Zhiheng Bian, Haoran Jiang, Taorui Liu, Yixin Sun, Han Peng, Xuesong Wang, Fanghao Lu, Chao Zhang, Fan Zhang, Qing Qin, Houjie Liang, Jianjun Li, Zhihao Wei, Yan Dong

**Affiliations:** ^1^ Department of Oncology and Southwest Cancer Center Southwest Hospital Third Military Medical University (Army Medical University) Chongqing 400038 China; ^2^ Chongqing Key Laboratory of Translational Research for Cancer Metastasis and Individualized Treatment Cancer Hospital Chongqing University Chongqing 400038 China; ^3^ Department of Stem Cell and Regenerative Medicine Southwest Hospital Third Military Medical University (Army Medical University) ChongQing 400038 China; ^4^ Brain Research Center and State Key Laboratory of Trauma Burns, and Combined Injury Third Military Medical University Chongqing 400038 China

**Keywords:** Colorectal carcinoma, CX3CL1, Immunosuppression, Regulatory T cell, SIRT1

## Abstract

Metabolic reprogramming of tumor cells dynamically reshapes the distribution of nutrients and signals in the tumor microenvironment (TME), affecting intercellular interactions and resulting in metabolic immune suppression. Increased glucose uptake and metabolism are characteristic of many tumors. Meanwhile, the progression of colorectal carcinoma (CRC) relies on lipid metabolism. Therefore, investigating the role of glucolipid metabolic reprogramming on tumor immunity contributes to identifying new targets for immune suppression intervention in CRC. Our previous work demonstrated that SIRT1 is the hub gene involved in glucolipid metabolic conversion in CRC. Here, it is found that upregulated SIRT1 in CRC cells increases Treg functionality by promoting the secretion of CX3CL1. The CX3CL1‐CX3CR1 signaling activated transcription factors SATB1 and BTG2, promoting the differentiation of TCF7^+^ Treg cells into functionally enhanced TNFRSF9^+^ Treg cells. Multiplex immunofluorescence (mIHC) analysis of a CRC tissue microarray confirmed the promoting effect of CX3CL1 on Treg infiltration. Additionally, the therapeutic efficacy of CX3CR1 inhibitor monotherapy and combination therapy is validated with the PD‐1 antibody in the humanized subcutaneous CRC mouse model. This study elucidates a potential mechanism that metabolic reprogramming of cancer cells collaborates with subsequent immunosuppression to promote CRC progression.

## Graphical abstract

This study discovers that SIRT1, a hub gene involved in glucolipid metabolic conversion in colorectal carcinoma (CRC), stimulates CX3CL1 secretion in CRC cells by activating FOXO1. The CX3CL1‐CX3CR1 signaling promotes the differentiation of TCF7^+^ regulatory T cells (Tregs) into an enhanced immunosuppressive TNFRSF9^+^ Treg phenotype. CRC cells exert metabolic immunosuppression on the tumor microenvironment via the SIRT1‐CX3CL1 axis.

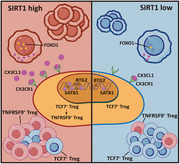

## 1 Introduction

Metabolic reprogramming and immune escape are two fundamental hallmarks of cancer. Nevertheless, they are not mutually exclusive. The dynamic variation of nutrients and signals within the TME drives the metabolic plasticity of cancer cells and facilitates the immune suppression of effector cells.^[^ ^1^ ^]^ In contrast to the traditional Warburg effect, the progression of CRC relies on lipid metabolism.^[^ ^2^ ^]^ However, whether glucolipid metabolic reprogramming affects the crosstalk between tumor and immune cells is elusive. Our previous studies revealed that the deacetylase SIRT1, which is widely distributed in the cytoplasm and nucleus, is a metabolic switch between glycolysis and fatty acid oxidation, its upregulation enables tumor cells to adapt to glucose deprivation and promotes tumor progression.^[^ ^3^ ^]^ Given that high SIRT1 expression in tumor cells can affect the expression and secretion of cytokines, thus influencing tumor growth and metastasis, further exploration of whether SIRT1 inhibits tumor immunity is warranted.^[^ ^4^ ^]^

Chemokines and their receptors have been a hot topic in tumor immunity research. The exocrine cytokine CX3CL1 (C‐X3‐C Motif Chemokine Ligand 1, also known as Fractalkine) has been shown to play an important role in the immune response. It is traditionally believed that CX3CL1 exerts an antitumor effect by recruiting CD8^+^ T cells. However, CX3CL1 participates in tumor invasion and metastasis in some tumors, leading to a poor prognosis.^[^ ^5^ ^]^ Recent studies have indicated that the interaction between tumor cells and other cells in the microenvironment mediated by CX3CL1, such as macrophages and mesenchymal stem cells, is related to developing an immunosuppressive environment.^[^ ^6^, ^7^ ^]^ Therefore, a comprehensive analysis of the mechanism of CX3CL1 production and function contributes to unraveling its effector network in TME.

Regulatory T cells (Tregs) are one of the main cell types that exert immunosuppressive effects in the tumor microenvironment. Their infiltration is closely associated with adverse clinical phenotypes such as treatment resistance, immune evasion, and metastasis. Recent studies have shown that tumor cells can recruit Treg cells to exert immunosuppressive effects and promote tumor progression by secreting certain cytokines, such as CCL17 and CCL20.^[^ ^8^, ^9^ ^]^ Notably, with the emergence of single‐cell RNA sequencing (scRNA‐seq) technology, it has become increasingly recognized that Treg cells exhibit heterogeneity in the tumor environment. A pan‐cancer analysis indicated that TNFRSF9^+^ Treg (TNF Receptor Superfamily Member 9, also known as 4‐1BB and CD137) cells are widely present in various types of tumor tissues and exert strong immunosuppressive effects.^[^ ^10^ ^]^ The transcription factor TCF‐1 (encoded by TCF7) plays a crucial role in the development of Treg cells, but in CRC, TCF‐1‐deficient Treg cells exhibit stronger immunosuppressive effects than WT Treg cells.^[^ ^11^ ^]^ These studies suggest that further elucidating the impact of cytokines on Treg phenotypes and functions is highly important.

In this study, we discovered that SIRT1 promotes the upregulation of CX3CL1 in CRC cells. CX3CL1, in turn, enhances the function of Tregs and suppresses antitumor immunity. Our findings suggested that tumor metabolic reprogramming and immune evasion cooperate to promote tumor progression.

## 2 Results

### 2.1 SIRT1 is Closely Related to the Immunosuppressive Tumor Microenvironment

**Figure 1:**
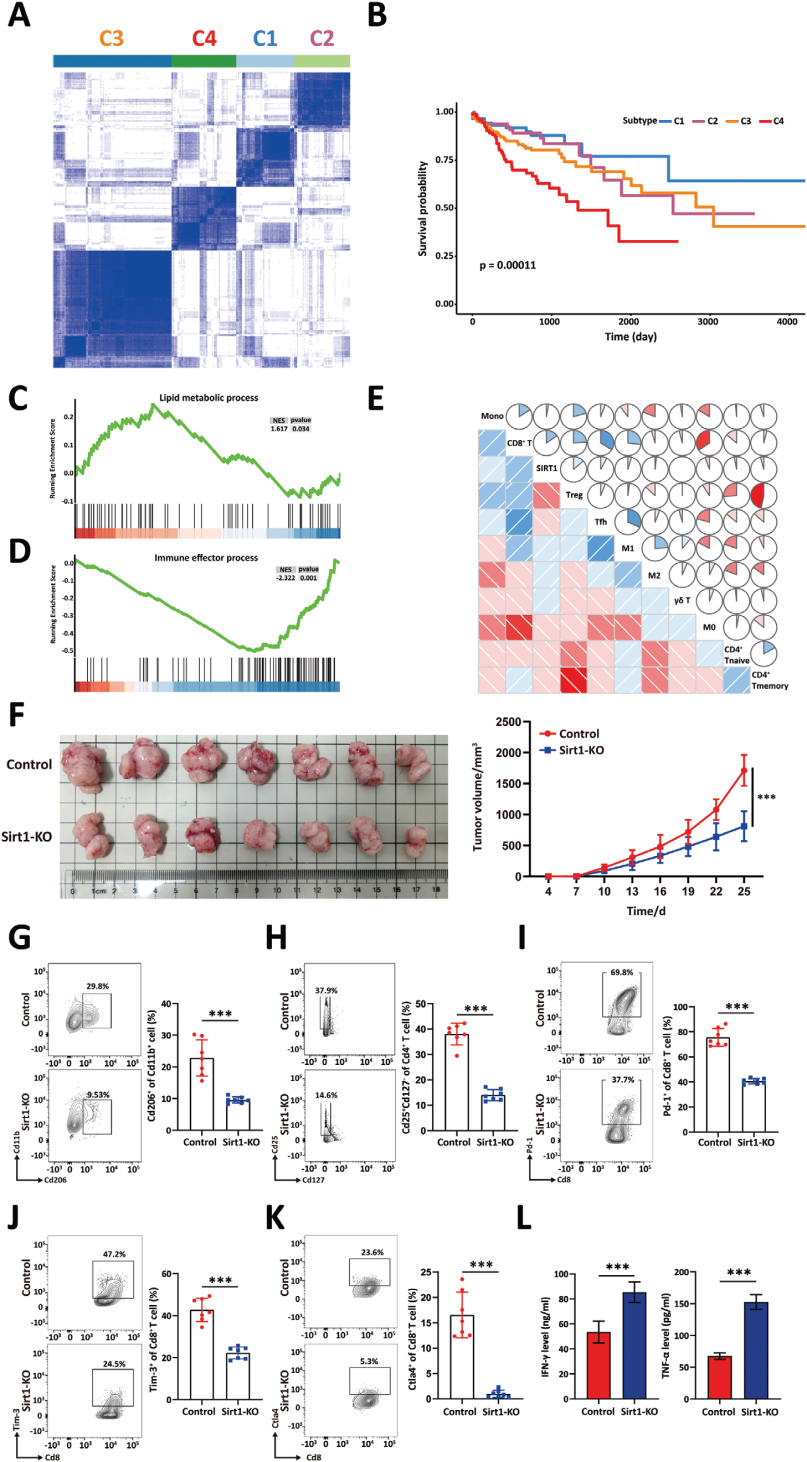
SIRT1 is closely related to the immunosuppressive tumor microenvironment. A) Consensus clustering of glucolipid metabolism‐related genes, which are significantly associated with prognosis, identifying four groups in the TCGA colorectal cancer cohort (TCGA‐COAD, *n* = 392). B) Kaplan‐Meier estimates of survival for patients with different glucolipid subtypes in the TCGA‐COAD cohort. C) The enrichment score of the “Lipid metabolic process” in C4 patients versus C1 patients was analyzed by GSEA using RNA‐seq data from the TCGA‐COAD cohort. D) Enrichment score of the “Immune effector process” in C4 patients versus C1 patients, analyzed by GSEA using RNA‐seq data of TCGA‐COAD cohort. E) The correlation of 10 main tumor‐infiltrating cell types with SIRT1 in CRC tissues. Red: positive correlation; blue: negative correlation. F) Representative gross appearance (left) of the subcutaneous allografts in the indicated groups. Tumor growth curves (right) of C57BL/6J mice inoculated with Sirt1‐KO or control MC38 allografts. Statistical significance was assessed by student *t*‐test of variance. ***p < 0.001. G) Flow cytometry analysis of the ratio of Cd206^+^ cells in Cd11b^+^ cells. *n* = 7, student *t*‐test of variance, ***p < 0.001. H) Flow cytometry analysis of the ratio of Cd25^+^Cd127^−^ cells in Cd4^+^ T cells. *n* = 7, student *t*‐test of variance, ***p < 0.001. I–K) Flow cytometry analysis of the ratio of Pd‐1^+^ cells in Cd8^+^ T cells (I), Tim‐3^+^ cells in Cd8^+^ T cells (J), and Ctla4^+^ cells in Cd8^+^ T cells (K). *n* = 7, student *t*‐test of variance, ***p < 0.001. L) Elisa of effector cytokines IFNγ (left), TNFα (right) from Sirt1‐KO or control MC38 allografts. *n* = 7, student *t*‐test of variance, ***p < 0.001.

The rapid energy provision and the production of key precursors for the biosynthesis of macromolecules make glycolysis the preferred energy supply method for cancer cells. However, metabolomic analysis of a large cohort study has found that the progression of CRC was highly dependent on lipid metabolism,^[^ ^2^ ^]^ suggesting that glucolipid metabolic reprogramming is involved in the development of CRC. To explore the specific roles of glycolysis and lipid metabolism in CRC progression, we conducted a univariate Cox regression analysis of all glycolysis‐ and lipid metabolism‐related genes in CRC samples from the TCGA database (*n* = 392). We identified 126 genes that were significantly associated with poor or prolonged survival in CRC (univariate Cox *p* < 0.05). The expression matrix of these genes served as the input for consensus clustering. Four groups were identified by consensus clustering: C1, C2, C3, and C4 (**Figure** **1**A). Kaplan‒Meier (K‒M) survival analysis indicated that the prognosis of the C4 group was significantly worse than that of the other groups (Figure 1B). Subsequently, we performed gene set enrichment analysis (GSEA) of the differentially expressed genes (DEGs) between patients in the C4 group and those in the C1 group. The results revealed a significant enrichment of lipid metabolism‐related gene sets and a markedly decreased enrichment of effector immune response‐related gene sets. At the same time, changes in glycolysis‐related pathways were not prominent (Figures 1C,D and S1A, Supporting Information). Correspondingly, we observed increased enrichment of pathways associated with macrophage differentiation and regulatory T‐cell differentiation related to immune suppression in patients in the C4 group (Figure S1B,C, Supporting Information). These results indicated that the enhanced lipid metabolism resulting from glucolipid metabolic reprogramming might promote the progression of CRC by reshaping the tumor immune microenvironment (TIME).

Our previous study revealed that SIRT1 was the hub that facilitated the metabolic shift from glycolysis to lipid metabolism in CRC cells.^[^ ^3^ ^]^ Here, we assessed whether SIRT1 shaped the tumor immune environment. First, we examined the correlation between SIRT1 expression and the levels of tumor‐infiltrating immune cells using the CIBERSORT algorithm.^[^ ^12^ ^]^ We found that SIRT1 was positively correlated with the infiltration of regulatory T cells (Tregs), but negatively correlated with CD8^+^ T cells (Figure 1E). Moreover, we discovered a positive correlation between SIRT1 mRNA expression and the expression of T‐cell exhaustion signature genes in CRC samples by performing Gene Expression Profiling Interactive Analysis (GEPIA) (Figure S1D, Supporting Information). Moreover, SIRT1 expression was positively correlated with markers of M2 macrophages and Tregs (Figure S1E,F, Supporting Information). These results suggested that SIRT1 participated in shaping the immunosuppressive tumor microenvironment.

Next, we established Sirt1 knockout (Sirt1‐KO) MC38 mouse CRC cell lines using lentiviral vectors and confirmed gene knockout efficiency by western blot analysis (Figure S1G, Supporting Information). Next, we constructed a subcutaneous allograft tumor model in C57BL/6 mice and generated tumor growth curves for the wild‐type (WT) MC38 and MC38‐Sirt1KO groups. When the living tumor volume in any tumor‐bearing mice exceeded 2000 mm^3^, all the mice were euthanized, and the tumor tissues were harvested and weighed. The experimental results indicated a significant inhibition of tumor progression in the Sirt1‐KO group (Figure 1F). Furthermore, we processed all tumor tissues by digesting them into single cells and then analyzed the infiltration of immune cells using flow cytometry. The results showed that the Sirt1‐KO group exhibited a significant decrease in the infiltration of immunosuppressive cells, such as M2‐type macrophages (Cd11b^+^Cd206^+^) and Treg cells (Cd4^+^Cd25^+^Cd127^−^), within the tumor tissue (Figure 1G,H). Meanwhile, we found that the expression level of SIRT1 did not affect the infiltration of myeloid‐derived suppressor cells (MDSCs, Cd11b^+^Gr1^+^) and dendritic cells (DCs, Cd11c^+^Cd86^+^) (Figure S1H,I, Supporting Information). Moreover, the expression of exhaustion‐related immune checkpoint markers (Pd‐1, Tim‐3, and Ctla4) was notably reduced in CD8^+^ T cells within the Sirt1‐KO cohort compared to the control group (Figure 1I–K). Correspondingly, we detected significantly increased levels of IFN‐γ and TNF‐α in the supernatants of the Sirt1‐KO single‐cell cultures (Figure 1L). These findings were consistent with previous bioinformatics analysis results, indicating that SIRT1 plays a crucial role in shaping the immunosuppressive tumor microenvironment.

### 2.2 SIRT1 Promotes the Expression of CX3CL1 in CRC Cells

**Figure 2:**
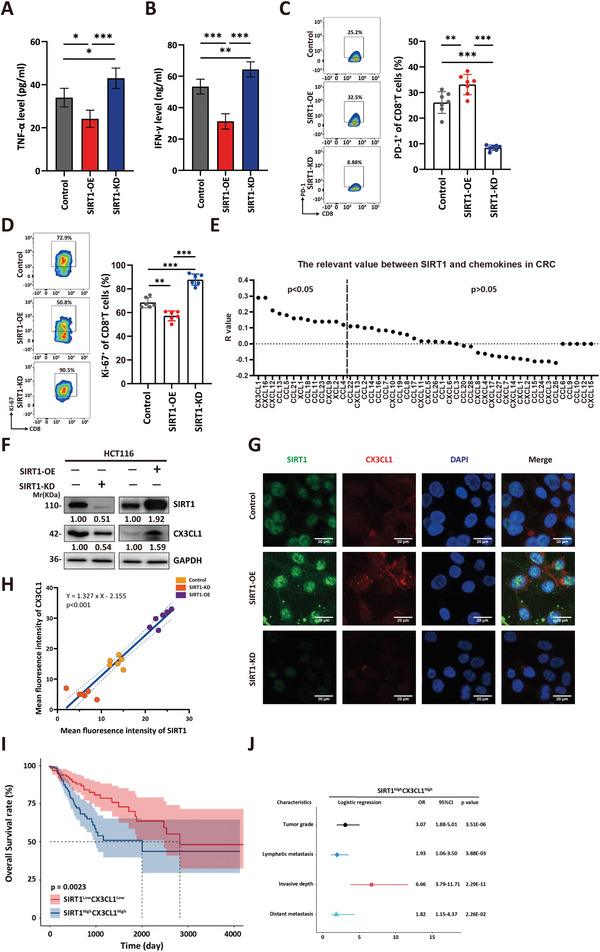
SIRT1 promotes the expression of CX3CL1 in colorectal tumor cells. A,B) Elisa of effector cytokines IFNγ (A), TNFα (B) in the supernatant of activated T cells co‐culture with indicated HCT116 cells. *n* = 6, one‐way ANOVA test of variance, *p <0.05, **p < 0.01, ***p < 0.001. C) Activated T cells were co‐cultured with indicated HCT116 cells. Flow cytometry analysis of the ratio of PD‐1^+^ cells in CD8^+^ T cells. *n* = 7, one‐way ANOVA test of variance, **p < 0.01, ***p < 0.001. D) Activated T cells were co‐cultured with indicated HCT116 cells. Flow cytometry analysis of the ratio of Ki67^+^ cells in CD8^+^ T cells. *n* = 6, one‐way ANOVA test of variance, **p < 0.01, ***p < 0.001. E) The correlation of SIRT1 with all chemokines, analyzed by GEPIA. F) The SIRT1 and CX3CL1 levels of SIRT1‐KD and control HCT116 (left) or SIRT1‐OE and control HCT116 (right) were assessed using immunoblotting. G,H) Confocal assay of SIRT1 (green) and CX3CL1 (red) in SIRT1‐OE, SIRT1‐KD, or control HCT116, and the fluorescence intensity was calculated by Image J software. Representative images of SIRT1‐KD, SIRT1‐OE, or control HCT116 (F) and correlation matching between SIRT1 and CX3CL1 (G) are shown. Scale bars, 20µm. I) Based on the median mRNA expression value, TCGA‐COAD samples were divided into two groups: SIRT1^Low^CX3CL1^Low^ and SIRT1^High^CX3CL1^High^. Kaplan‐Meier analysis of the overall survival rate of patients in different groups. Red line: SIRT1^Low^CX3CL1^Low^; Blue line: SIRT1^High^CX3CL1^High^. J) Multivariate logistic regression analysis of odds ratio (ORs) of different clinic‐pathological characteristics showed that compared with SIRT1^Low^CX3CL1^Low^ profile, SIRT1^High^CX3CL1^High^ profile had a higher risk of large tumor grade, invasive depth, and metastasis.

We subsequently evaluated the impact of alterations in SIRT1 expression levels within tumor cells on tumor immunity. Previous studies have highlighted the pivotal role of chemokines in facilitating the interplay between tumor cells and immune cells, orchestrating the generation and recruitment of immune cells that foster a tumor‐promoting microenvironment.^[^ ^13^ ^]^ Therefore, we hypothesized that SIRT1 might be associated with the secretion of certain chemokines. To test this hypothesis, we first constructed overexpression and knockdown plasmids for SIRT1, and transduced them into two human CRC cell lines, HCT116 and SW480, to obtain cell models with either SIRT1 overexpression (OE) or SIRT1 knockdown (KD). Subsequently, we isolated T cells from human peripheral blood mononuclear cells (PBMCs) and activated them with anti‐CD3, anti‐CD28 antibodies, and IL‐2 for 72 h. Then, we seeded tumor cells in the basolateral chamber of a transwell system and added the activated T cells to the apical chamber (Figure S2A, Supporting Information). The coculture mixture was maintained for 72 h, after which cell culture supernatants were collected to assess the effector cytokines IFN‐γ and TNF‐α expression in the different groups (**Figures** **2**A,B and S2B,C, Supporting Information). We also evaluated the expression levels of PD1 and Ki67 in CD8^+^T cells to assess their exhaustion state (Figures 2C,D and S2C,D, Supporting Information). Compared to those cultured with tumor cells from the control group, T cells cocultured with tumor cells from the SIRT1‐OE group exhibited reduced IFN‐γ and TNF‐α production, decreased Ki‐67 expression, and increased PD‐1 expression. Coculture of T cells with SIRT‐KD tumor cells had the opposite effect. These experiments indicated that SIRT1 in tumor cells could suppress anti‐tumor immunity through paracrine signaling. Additionally, we found that the expression level of SIRT1 was negatively correlated with the apoptosis rate of tumor cells (Figure S2E,F, Supporting Information). These data suggested that the co‐culture system served as an excellent in vitro model for investigating the one‐way influence of tumor cells on T cells.

Next, we analyzed the correlation between SIRT1 and the expression of all chemokines using GEPIA in the TCGA database. The results demonstrated that among chemokines, CX3CL1 was the most strongly correlated with SIRT1 in CRC (Figure 2E). Subsequently, we performed immunoblotting and immunofluorescence analyses to confirm the positive correlation between the expression levels of SIRT1 and CX3CL1 further (Figure 2F–H). We then assessed whether SIRT1 and CX3CL1 could be combined to indicate CRC prognosis. CRC samples from the TCGA database were divided into high and low‐expression groups based on the median expression levels of SIRT1 and CX3CL1, respectively. The intersection of expression groups for both genes revealed that 188 samples had the same expression patterns for SIRT1 and CX3CL1 (96 samples had high expression of both SIRT1 and CX3CL1, and 92 samples had low expression of both SIRT1 and CX3CL1). Survival analysis of these samples revealed that patients with a SIRT1^High^CX3CL1^High^ profile had a significantly lower overall survival rate than those with a SIRT1^Low^CX3CL1^Low^ profile (Figure 2I). Moreover, the upregulation of SIRT1 and CX3CL1 was significantly correlated with several aggressive clinicopathological features of CRC, including advanced tumor stage, increased lymphatic and distant metastasis rates, and increased tumor invasion depth (Figure 2J). These findings suggested that the SIRT1‐CX3CL1 axis was critical in promoting the progression and spread of CRC and could serve as a prognostic biomarker for identifying patients with a high likelihood of poor outcomes.

### 2.3 SIRT1 Activates FOXO1 to Promote CX3CL1 Expression

**Figure 3:**
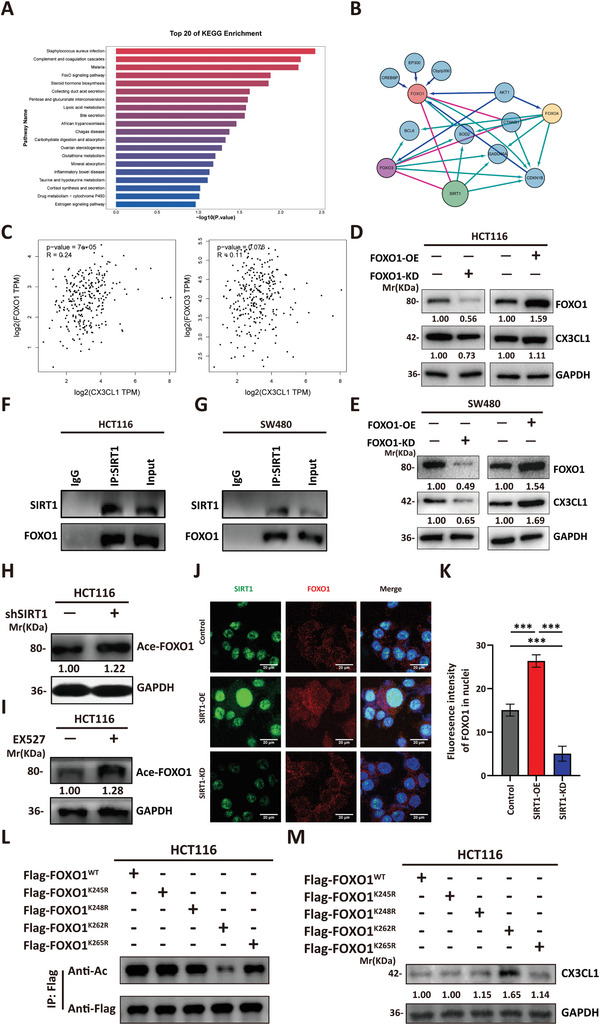
SIRT1 activates FOXO1 to promote CX3CL1 expression. A) The upregulated DEGs of SIRT1‐OE relative to SIRT1‐WT HCT116 cells were subjected to KEGG pathway enrichment analysis. The top 20 KEGG pathways with the most significant *p*‐values were displayed. B) Screening graph showing the relationship between SIRT1 and FOXO family members using cBioPortal in CRC samples. C) GEPIA analysis of the correlation between CX3CL1 and FOXO1 or FOXO3. D,E) FOXO1 and CX3CL1 levels of FOXO1‐KD, FOXO1‐OE, and control HCT116 (D) or SW480 (E) were assessed using immunoblotting. F,G) SIRT1 immune complexes were immunoprecipitated from HCT116 (F) or SW480 (G) cells and subjected to immunoblotting of FOXO1. H,I) Acetyl level of FOXO1 in SIRT1‐KD (H) or SIRT1 inhibitor EX527 (20 µM, 48 h) treated HCT116 cells (I) was assessed using immunoblotting. J,K) Confocal assay of SIRT1 (green) and FOXO1 (red) in SIRT1‐OE, SIRT1‐KD, or control HCT116 and the fluorescence intensity of FOXO1 in nuclear was calculated by Image J software. Representative images of SIRT1‐KD, SIRT1‐OE, or control HCT116 (J) and the statistical analysis of nuclear fluorescence intensity of CX3CL1 (K) are shown. One‐way ANOVA test of variance, ***p < 0.001. Scale bars, 20µm. L) The indicated plasmids were transfected into HCT116 cells, and the acetylation of FOXO1^WT^ and mutants was measured by immunoblotting. (M) CX3CL1 levels of cells in (L) were assessed using immunoblotting.

Next, we explored the mechanisms by which SIRT1 enhances the expression of CX3CL1. First, we performed RNA sequencing (RNA‐seq) on WT and SIRT1‐OE HCT116 cells. To identify signaling pathways that SIRT1 might directly regulate, we conducted a Kyoto Encyclopedia of Genes and Genomes (KEGG) analysis of the DEGs in the SIRT1‐OE group. We found significant enrichment of the FoxO signaling pathway in the SIRT1‐OE cells (**Figures** **3**A and S3A, Supporting Information). The FoxO transcription factor family consists of four members: FOXO1, FOXO3, FOXO4, and FOXO6. Screening based on the cBioPortal website revealed that FOXO1 and FOXO3 exhibited the strongest correlation with SIRT1 (Figure 3B). GEPIA analysis indicated that the expression of CX3CL1 was significantly positively correlated with that of FOXO1 but not with that of FOXO3 in CRC patients (Figure 3C). Moreover, the expression of SIRT1 was positively correlated with that of FOXO1 in various gastrointestinal cancers (Figure S3B, Supporting Information). To further validate the FOXO1 function, we separately performed FOXO1 or FOXO3 overexpression or knockdown experiments in HCT116 and SW480 cells and detected the expression of CX3CL1 in the culture supernatant via ELISA (Figure S3C,D, Supporting Information) and western blot (Figures 3D,E and S3F,G, Supporting Information). We observed that FOXO1 significantly affects the level of CX3CL1, while FOXO3 has a relatively minor impact on the level of CX3CL1 (Figure S3E,H Supporting Information). Moreover, treatment of CRC cells with a FOXO1‐specific inhibitor (AS1842856) significantly suppressed the secretion of CX3CL1 (Figure S3I, Supporting Information). These data indicated that SIRT1 promoted CX3CL1 secretion by regulating FOXO1.

Then, we used coimmunoprecipitation experiments to validate the endogenous SIRT1‐FOXO1 interaction in CRC cell lines (Figure 3F,G). Given that SIRT1 primarily functions as a deacetylase within cells, we treated CRC cells with shSIRT1 or the SIRT1‐specific inhibitor EX527. Both treatments resulted in elevated acetylation levels of the transcription factor FOXO1 (Figures 3H,I and S3J,K Supporting Information). This finding indicates that SIRT1 acts as a deacetylase for FOXO1. Next, we assessed whether the acetylation level of FOXO1 affected its nuclear localization. We performed immunofluorescence staining to evaluate the subcellular localization of FOXO1 in SIRT1‐WT, SIRT1‐KD, and SIRT1‐OE HCT116 cells. The results showed that deacetylation promoted the nuclear accumulation of FOXO1, while acetylated FOXO1 exhibited significantly reduced stability within the nucleus (Figure 3J,K). To directly examine the impact of FOXO1 acetylation status on its function, we constructed five Flag‐tagged plasmids: FOXO1^K245R^‐Flag, FOXO1^K248R^‐Flag, FOXO1^K262R^‐Flag, and FOXO1^K265R^‐Flag and FOXO1^WT^‐Flag, to simulate the deacetylated state of FOXO1. We knocked down the endogenous FOXO1 in tumor cells to minimize its impact on the experimental outcomes (Figure S3L, Supporting Information). Then we transfected five plasmids and found that each of the mutated plasmids reduced the acetylation levels of FOXO1, with the arginine mutation at position 262 showing the most pronounced effect (Figure 3L). Subsequently, through western Blot detection, we found that the expression of CX3CL1 is negatively correlated with the acetylation levels of FOXO1 (Figure 3M). These results suggested that SIRT1 stabilizes FOXO1 in the nucleus via deacetylation, allowing it to exert its transcriptional effects. In summary, the above experiments demonstrated that SIRT1 deacetylates FOXO1 to promote the expression of CX3CL1 in CRC cells.

### 2.4 CX3CL1 Promotes Immune Suppression by Mediating the Function of Treg Cells

**Figure 4:**
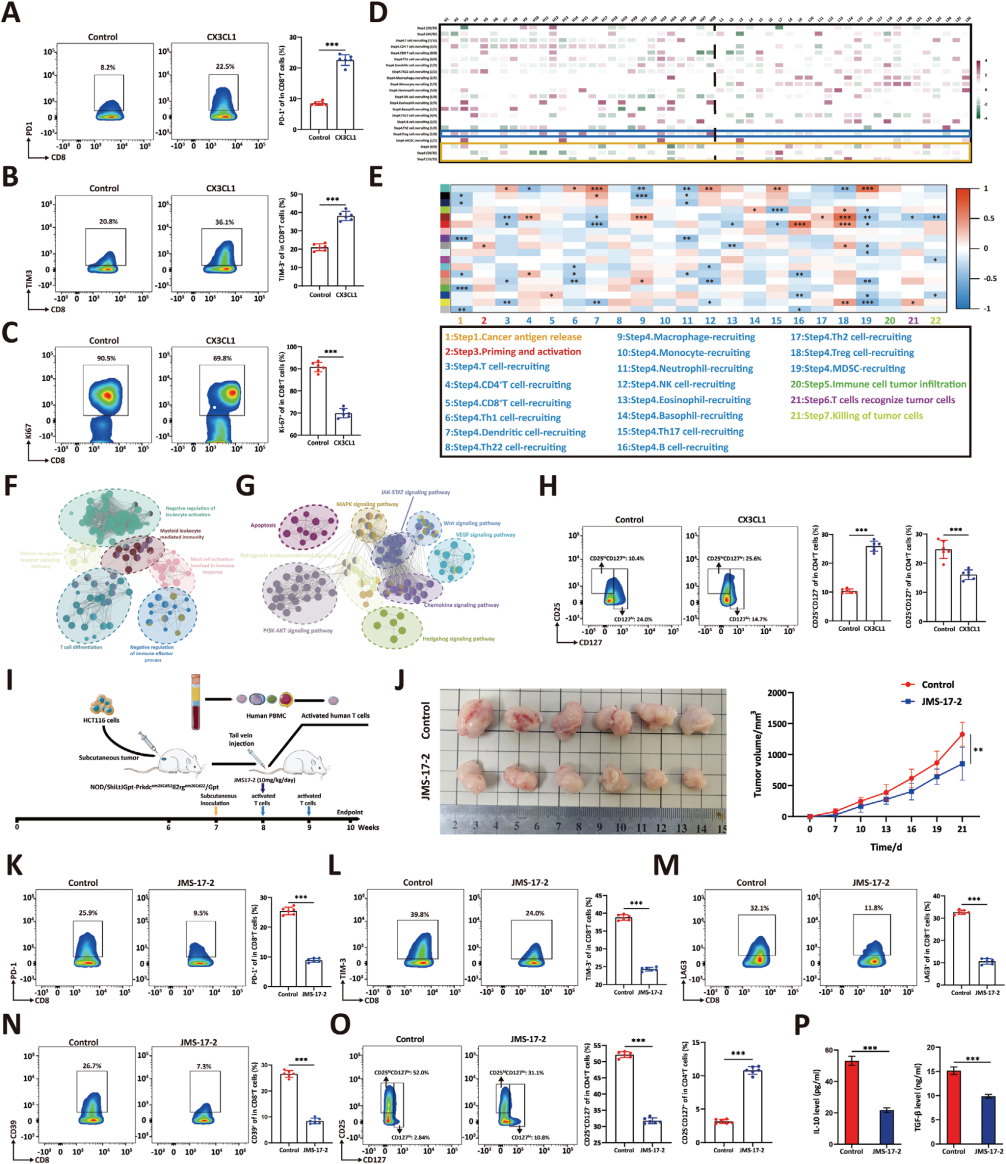
CX3CL1 promotes immune suppression by mediating the function of Treg cells. A–C) Activated T cells co‐cultured with HCT116 cells received indicated treatment (CX3CL1, 200 ng ml^−1^, 48 h), and flow cytometry analysis of the ratio of PD‐1^+^ cells in CD8^+^ T cells (A); the ratio of TIM‐3^+^ cells in CD8^+^ T cells (B); the ratio of Ki67^+^ cells in CD8^+^ T cells (C). *n* = 6, student *t*‐test of variance, ***p < 0.001. D) The seven‐step cancer immunity cycle scores of CX3CL1^High^ and CX3CL1^Low^ CRC samples from the TCGA database. E) Using the CICS as a phenotype, upregulated DEGs of CX3CL1High versus CX3CL1Low CRC samples were used to construct a WGCNA coexpression network; each color represents a type of module. The heatmap shows the correlation of each module with CICS. Red represents a positive correlation, while blue represents a negative correlation. *p <0.05, **p < 0.01, ***p < 0.001. F) The ClueGO plug‐in was used to perform GO enrichment for the core genes in brown modules. Each node represents a biological process; nodes with the same color have similar functions. Node size shows the significance of enrichment. G) The ClueGO plug‐in was used to perform KEGG enrichment for the core genes in brown modules. Each node represents a signaling pathway; nodes with the same color have similar functions. Node size shows the significance of enrichment. H) Activated T cells co‐cultured with HCT116 cells received indicated treatment, and flow cytometry analysis of the ratio of CD25^+^CD127^−^ (Treg) cells and CD25^−^CD127^+^ (memory) cells in CD4^+^ T cells. *n* = 6, student *t*‐test of variance, ***p < 0.001. I) Schematic diagram of constructing a humanized mouse subcutaneous transplant tumor model. J) NCG mice with subcutaneous xenografts (10^6^ HCT116 cells) have tail vein injected CD4^+^/CD8^+^ mixed T cells. All mice were divided into two groups and received indicated treatment, and tumor tissues were extracted at the experimental endpoint (left). The tumor size was measured every three days using calipers to plot the tumor growth curve (right). Statistical significance was assessed by student *t*‐test of variance. *n* = 6, **p < 0.01. K–N) Tumor tissues in (J) were digested into single cells, and flow cytometry analysis of the ratio of PD‐1^+^ cells in CD8^+^ T cells (K); the ratio of TIM‐3^+^ cells in CD8^+^ T cells (L); the ratio of LAG‐3^+^ cells in CD8^+^ T cells (M) and the ratio of CD39^+^ cells in CD8^+^ T cells (N). *n* = 6, student *t*‐test of variance, ***p < 0.001. O) Tumor tissues in (J) were digested into single cells, and flow cytometry analysis of the ratio of CD25^+^CD127^−^ (Treg) cells and CD25^−^CD127^+^ (memory) cells in CD4^+^ T cells. *n* = 6, student *t*‐test of variance, ***p < 0.001. P) TGF‐β (left) and IL‐10 (right) levels of JMS‐17‐2 treated or control HCT116 xenografts (single cell culture supernatant after tissue lysis) were assessed by ELISA assay. *n* = 6, student *t*‐test of variance, ***p < 0.001.

Next, we investigated the potential role of CX3CL1 in developing an immunosuppressive microenvironment. First, GEPIA analysis revealed a positive correlation between the expression of CX3CL1 and T‐cell exhaustion markers in human CRC samples (Figure S4A, Supporting Information). Then, we cocultured HCT116 cells with activated T cells in transwell chambers as previously described. We found that the exogenous addition of CX3CL1 significantly increased the expression of exhaustion‐related markers on CD8^+^ T cells (**Figure** **4**A,B). Recent studies have indicated that early exhausted T cells exhibit strong proliferative capacity and retained tumor reactivity. In contrast, terminally exhausted T cells exhibit reduced proliferative capacity and decreased effector cytokines production.^[^ ^14^ ^]^ Correspondingly, we found that the exogenous addition of CX3CL1 reduced the proliferative capacity and suppressed the production of TNF‐α and IFN‐γ by CD8^+^ T cells (Figures 4C and S4B,C, Supporting Information). These results suggest that CX3CL1 regulates T‐cell exhaustion.

To investigate the mechanism by which CX3CL1 mediates T‐cell exhaustion, we calculated the cancer‐immunity cycle score (CICS) (http://biocc.hrbmu.edu.cn/TIP/)^[^ ^15^ ^]^ using mRNA expression data for the 28 samples with the highest CX3CL1 expression and the 28 samples with the lowest CX3CL1 expression among TCGA CRC samples. The results showed that the high‐CX3CL1‐expression group had a significantly greater infiltration level of Tregs and lower T‐cell effectiveness (steps 5–7) in tumor tissue than did the low‐expression group (Figure 4D). These data suggested that the enhanced immunosuppressive phenotype of the CX3CL1 high‐expression group might be related to Tregs. Next, we identified the DEGs upregulated in the CX3CL1 high‐expression group compared to the low‐expression group and used the CICS as a clinical marker for weighted gene co‐expression network analysis (WGCNA). We identified 18 co‐expressed gene modules and performed a correlation analysis between these modules and the CICS phenotype. We found that most modules were related to Treg recruitment, with ten modules showing positive correlations and 5 being significantly positively correlated (Figure 4E). Furthermore, we conducted GO and KEGG analyses of the genes in the brown module, which was most closely associated with Treg infiltration. GO analysis revealed that these genes were enriched in processes related to immunosuppression, such as “negative regulation of leukocytes,” “negative regulation of immune effector process,” and “myeloid leukocyte‐mediated immunity” (Figure 4F). On the other hand, KEGG analysis revealed that these genes were enriched in classical pathways that activate Treg function, including the “JAK‐STAT signaling pathway” and “MAPK signaling pathway” (Figure 4G). We also found a significant positive correlation between the expression of CX3CL1 and that of the key transcription factor FOXP3, which regulates Treg function, as well as between the expression of the Treg effector molecules IL‐10 and TGFβ in human CRC tissues via GEPIA (Figure S4D, Supporting Information). These results suggest that CX3CL1 helps tumor cells establish an immunosuppressive microenvironment by promoting the infiltration and enhancing the function of Tregs.

We then proceeded to validate the effect of CX3CL1 on Treg cells directly via in vitro experiments. T cells isolated from PBMCs were activated with anti‐CD3, anti‐CD28 antibodies, and IL‐2. Then activated T cells were cocultured with exogenously added CX3CL1 for 48 h. The results showed that, compared to those in the control group, the proportion of Treg cells (CD4^+^CD25^+^CD127^−^) was significantly higher, and the proportion of memory T cells (CD4^+^CD25^−^CD127^+^) was significantly lower in the CX3CL1‐treated group (Figure 4H). Although CX3CL1 enhances the function of Treg cells, we could not determine whether the increase in T‐cell exhaustion induced by CX3CL1 is due to its effect or mediated by Treg cells. To address this issue, we sorted *ex vivo*‐activated T cells into CD4^+^ T cells and CD8^+^ T cells. Compared to adding CX3CL1 only in CD8^+^ T cells, adding CX3CL1 in mixed CD4^+^ T and CD8^+^ T cells significantly increased the expression levels of exhaustion markers (Figure S4E,F, Supporting Information) and attenuated proliferative capacity (Figure S4G, Supporting Information) on CD8^+^ T cells.

We further validated that CX3CL1 promotes T‐cell exhaustion by regulating Treg function in humanized mice subcutaneously engrafted with CRC cells. Specifically, one week after the injection of HCT116 cells into the flanks of NOD/ShiLtJGpt (NCG) mice to induce subcutaneous tumor formation, in vitro activated CD8^+^ T cells (1  ×  10^7^ per mouse) or mixed CD4^+^ T cells (5 × 10^6^ per mouse) and CD8^+^ T cells (5  ×  10^6^ per mouse) cells from the same healthy donor were injected via the tail vein every week (Figure 4I). We also treated mice injected with CD8^+^ T cells or mixed T cells with the CX3CL1‐specific inhibitor JMS‐17‐2 (10mg kg^−1^ per day). We discovered that in mice intravenously injected with a mixture of CD4^+^ T and CD8^+^ T cells via the tail vein, JMS‐17‐2 significantly inhibited tumor progression (Figure 4J). However, the antitumor effect of JMS‐17‐2 was not pronounced in mice that were solely administered CD8^+^ T cells (Figure S4H, Supporting Information). At the endpoint of the in vivo experiment in which mice were injected with mixed T cells, we euthanized all the mice, extracted the tumors, and digested them into single cells for flow cytometric analysis. Firstly, we evaluated the infiltration of T cells in tumor tissues and found that JMS‐17‐2 could significantly increase the level of T cell infiltration (Figure S4I, Supporting Information). The proportion of T cells was between 18%‐25%, which is essentially consistent with the abundance of T cell infiltration in surgically resected tissues from patients.^[^ ^16^ ^]^ Next, we found that applying JMS‐17‐2 treatment could significantly increase the proliferative capacity of CD8^+^ T cells, suggesting a stronger tumor reactivity (Figure S4J, Supporting Information). Notably, the administration of JMS‐17‐2 significantly alleviated CD8^+^ T cell exhaustion in the mice, as evidenced by the decreased expression of exhaustion markers such as PD‐1, TIM‐3, LAG‐3, and CD39 (Figure 4K–N). Correspondingly, we found that JMS‐17‐2 treatment reduced Treg infiltration and a significant increase in the proportion of memory T cells (Figure 4O). We also conducted ELISA tests on the primary cell supernatant and found that IL‐10 and TGFβ levels were significantly decreased in the JMS17‐2‐treated group, indicating diminished Treg function (Figure 4P). To further validate the immunomodulatory effects of CX3CL1‐CX3CR1 signaling blockade, we administered neutralizing antibodies targeting CX3CL1 (Quetmolimab, 200 µg  per mouse/3 days) to assess the immunosuppressive effects of CX3CL1‐CX3CR1 signal blocking in humanized mouse models injected with a mixture of CD4^+^ T and CD8^+^ T cells. We observed that, compared to the control group, Quetmolimab treatment exhibited a significant anti‐tumor effect (Figure S5A, Supporting Information). The immune microenvironment analysis revealed that Quetmolimab treatment significantly reduced the expression of exhaustion markers (Figure S5B,C, Supporting Information) and increased the tumor reactivity of CD8^+^ T cells (with elevated expression of Ki67, TNF, and IFN) (Figure S5D–F, Supporting Information), indicating a reduction in CD8^+^ T cell exhaustion. Correspondingly, the blockade of CX3CL1 led to a decrease in the infiltration of Treg cells (Figure S5G, Supporting Information). Taken together, these findings indicated that CX3CL1 enhanced the function of Treg cells to suppress anti‐tumor immunity.

### 2.5 CX3CL1 Enhances the Function of Treg Cells by Promoting the TNFRSF9 Phenotype

**Figure 5:**
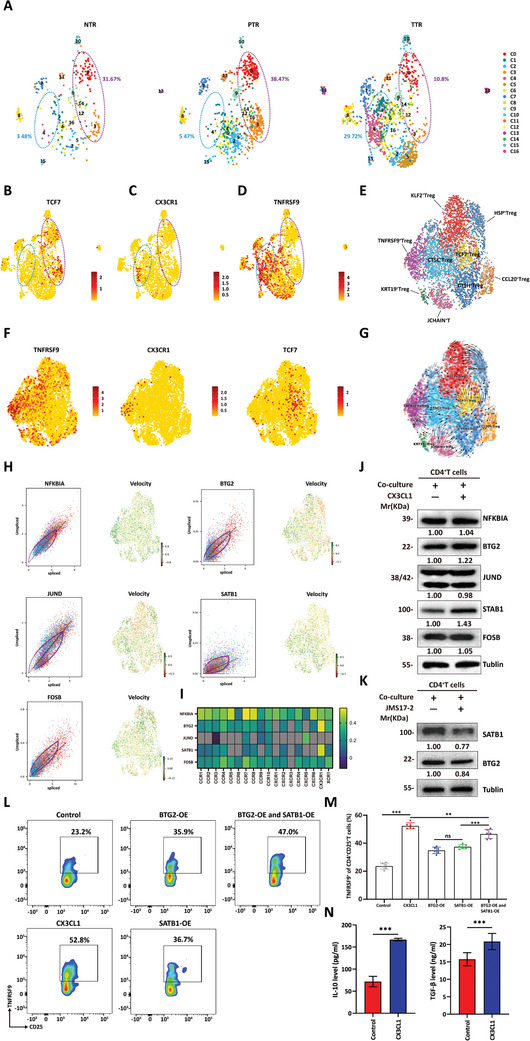
CX3CL1 enhances the function of Treg cells by promoting the TNFRSF9 phenotype. A) UMAP plot based on the scRNA‐seq data (GSE108989) of Treg cells sourced from peripheral blood, adjacent normal tissue, and cancer tissue. Create visual representations that reflect the origin of the samples. Determine and quantify the percentages of cells within Clusters 0, 3 (encircled by the purple dotted line) and Cluster 1, 4 (encircled by the blue dotted line) for each sample category in relation to the overall number of cells. NTR, Tregs from adjacent normal colorectal tissues; PTR, Tregs from peripheral blood; TTR, Tregs from CRC. B–D) Feature plots of indicated genes across all Treg subclusters in (A). E) scRNA‐seq of CD45^+^ immune cells extracted from resected tumor tissues of three primary‐care CRC patients. UMAP plot of Tregs (CD4^+^FOXP3^+^, *n* = 4628), identifying nine distinct subgroups. Each subgroup was named by marker genes calculated using the Seurat package. F) Feature plots of indicated genes across all Treg subclusters in (E). G) UMAP and extrapolated future state of cells (overlaid arrows) based on RNA velocity. H) Putative driver genes of TNFRSF9^+^ Treg are identified by high likelihoods. Phase portraits (left) and expression dynamics (right, green represents upregulation, while red represents downregulation) for these driver genes characterize their activity. I) Heatmap shows the GEPIA analysis of the correlation between putative driver genes in (H) and all chemokine receptors. J) The expression level of putative transcription factors in CX3CL1 treated or control CD4^+^ T cells was assessed using immunoblotting. K) The expression level of BTG2 and SATB1 in CX3CL1 inhibitor JMS‐17‐2 treated or control CD4^+^ T cells was assessed using immunoblotting. L,M) Co‐culturing CD4^+^ T cells from different groups, as illustrated, with HCT116 cells. The ratio of TNFRSF9^+^ cells in CD25^+^ T cells was analyzed by flow cytometry. *n* = 6, student *t*‐test of variance, **p < 0.01, ***p < 0.001. (N) Exogenous addition of CX3CL1 significantly increased the IL‐10 (left) and TGF‐β (right) levels of CD4^+^ T cells co‐cultured with HCT116. *n* = 6, student *t*‐test of variance, ***p < 0.001.

To further investigate the role of CX3CL1 signaling in Treg cells, we first examined the expression of CX3CL1 and its unique receptor CX3CR1 in different Treg populations. We examined scRNA‐seq data (GSE108989) from tissues of 12 CRC patients and found that Treg cells from PBMCs and adjacent normal tissue hardly expressed CX3CR1. In contrast, a subset of Treg cells infiltrating the tumor tissue exhibited high expression of CX3CR1 (Figure S6A, Supporting Information). Next, we integrated all pairs of Treg cell samples. The UMAP analysis revealed distinct origins of Treg cell clusters: Cells in Clusters 0 and 3 were primarily from PBMCs and adjacent normal tissues, making up 31.67% and 38.47% of these subgroups, respectively, with only 10.8% from CRC tissues. In contrast, Clusters 1 and 4 were mainly tumor tissue‐infiltrating Treg cells, with 29.72% from CRC, while contributions from adjacent tissues and PBMCs were minimal at 3.48% and 5.47%, respectively (**Figures** **5**A and S6B, Supporting Information). The above results indicated that the phenotype of tumor‐infiltrating Treg cells significantly differed from that of Tregs derived from PBMCs or adjacent normal tissues.

A previous study reported that TCF‐1‐deficient Treg cells strongly suppressed T‐cell proliferation and cytotoxicity.^[^ ^11^ ^]^ Correspondingly, Cluster 0 and Cluster 3 Treg cells exhibited high TCF7 expression (encoding the TCF1), while Cluster 1 and Cluster 4 Treg cells barely expressed TCF7 (Figure 5B). Moreover, we found that CX3CR1 was primarily expressed in Treg cells from Cluster 4 (Figure 5C). Notably, TNFRSF9^+^ Tregs are a subset that has been demonstrated to play a crucial immunosuppressive role in various tumors.^[^ ^10^ ^]^ Our analysis showed that Treg cells in both Cluster 1 and Cluster 4 were in the TNFRSF9^+^ Treg subset (Figure 5D). Because of the overlap between CX3CR1^+^ cells and TNFRSF9^+^ cells, these results suggested that the CX3CL1‐CX3CR1 signaling pathway might be involved in shaping the TNFRSF9^+^ Treg phenotype. Lipid synthesis and metabolism are closely associated with Treg differentiation and functional enhancement.^[^ ^17^ ^]^ We identified DEGs between Treg cells in Cluster 4 and those in Clusters 0 and 3, and subsequently conducted GSEA. The results indicated that lipid biosynthetic and lipid metabolism processes are more active in Tregs from Cluster 4 (Figure S6C, Supporting Information). Moreover, genes associated with the “receptor signaling pathway via JAK‐STAT” pathway were significantly enriched in Cluster 4 (Figure S6D, Supporting Information). The above data suggested that CX3CL1‐CX3CR1 signaling might promote Treg function.

To explore the impact of CX3CL1‐CX3CR1 signaling on the phenotype of Tregs, we performed scRNA‐seq on CD45^+^ immune cells sourced from three resected CRC samples. We then integrated and clustered the CD4^+^FOXP3^+^ cells (*n* = 4628) and identified nine subgroups after batch effect removal (Figure 5E). Consistent with the above results, we observed similar expression patterns of CX3CR1 and TNFRSF9, and the CX3CR1^+^ cells did not express TCF7 (Figure 5F). Subsequent RNA velocity analysis of the scRNA‐seq data indicated that TCF7^+^ Treg cells served as the starting point for the differentiation of various phenotypes of Treg cells in the tumor microenvironment. TNFRSF9^+^ Treg cells were identified as a terminal differentiation phenotype (Figure 5G). Next, by individual gene dynamics screening of transcription factors, we identified five potential transcription factors that correlated most strongly with the differentiation of TCF7^+^ Treg cells to TNFRSF9^+^ Treg cells: NFKBIA, BTG2, JUND, SATB1, and FOSB (Figure 5H). To further clarify the transcription factors associated with the CX3CL1‐CX3CR1 signaling pathway, we calculated the correlation of these transcription factors with the expression of all chemokine receptors in CRC samples from the TCGA database. The results indicated that CX3CR1 strongly correlates with BTG2 and SATB1 (Figure 5I). Next, we activated T cells isolated from PBMCs and cocultured them with HCT116 cells after sorting the CD4^+^ T cells. We observed that the exogenous addition of CX3CL1 increased the expression of SATB1 and BTG2 in CD4^+^ T cells, while its impact on the other three transcription factors was minimal (Figures 5J and S6E, Supporting Information). Simultaneous JMS17‐2 treatment reduced the expression of SATB1 and BTG2 (Figures 5K and S6F, Supporting Information). To further validate the promoting effect of BTG2 and SATB1 on the TNFRSF9^+^ Treg phenotype, we constructed lentiviruses for overexpressing these two transcription factors. T cells activated by anti‐CD3, anti‐CD28, and IL‐2 were transfected with empty vector, BTG2‐OE, SATB1‐OE, and a combination (BTG2‐OE+SATB1‐OE) lentivirus. The overexpression of transcription factors was verified by western blotting (Figure S6G, Supporting Information). After sorting CD4^+^T cells by flow cytometry, we co‐cultured them with tumor cells according to the illustrated treatment. We then detected the proportion of TNFRSF9^+^ Treg cells after 72 h. The results indicated that overexpressing BTG2 and SATB1 significantly increased the differentiation of the TNFRSF9^+^ Treg phenotype, and the combined overexpression achieved an effect comparable to that of CX3CL1 stimulation (Figures 5L,M and S6H,I, Supporting Information). These results suggested that BTG2 and SATB1 are downstream effector molecules of the CX3CL1‐CX3CR1 signaling pathway. Finally, we co‐cultured CD4^+^ T cells with tumor cells for 48 h and found that the exogenous addition of CX3CL1 significantly promoted the secretion of IL10 and TGFβ (Figures 5N and S6J, Supporting Information). Therefore, these results indicated that the CX3CL1‐CX3CR1 signaling pathway facilitates the induction of the TNFRSF9^+^ Treg phenotype by activating BTG2 and SATB1.

### 2.6 The Clinical Relevance of CX3CL1 Signaling

**Figure 6:**
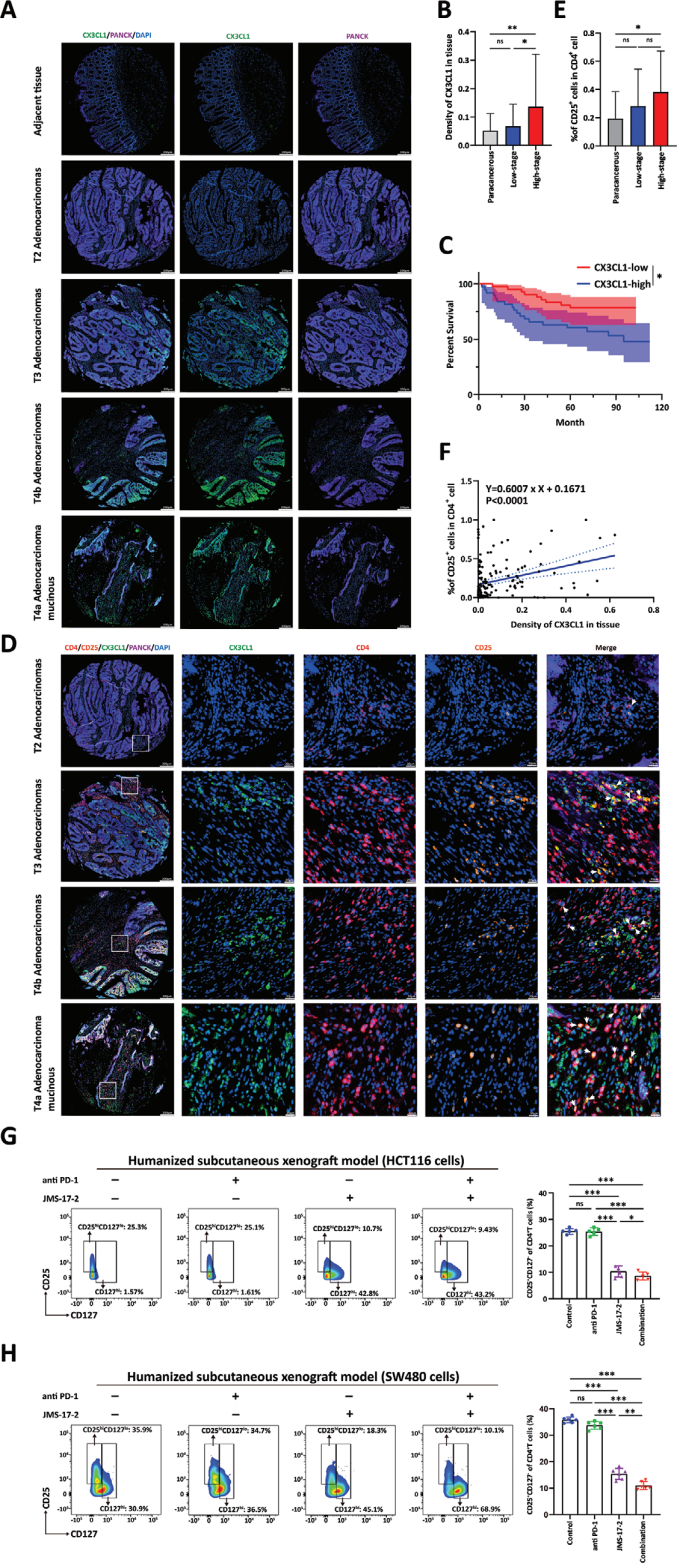
The clinical relevance of CX3CL1 signaling. A) Representative mIHC images of different‐stage tumor tissue showing the expression level of CX3CL1 in the PANCK^+^ tumor area. Green, CX3CL1; Purple, PANCK; Blue, DAPI. Scale bars, 200 µm. B) Statistics analysis of the ratio of CX3CL1^+^/PANCK^+^ in the adjacent normal, low‐grade tumor, and high‐grade tumor tissues in mIHC‐stained tissue microarrays. One‐way ANOVA test of variance, ns, non‐significant, *p < 0.05, **p < 0.01. C) All samples were divided into two groups, CX3CL1^High^ and CX3CL1^Low^, based on the fluorescence intensity of CX3CL1. Kaplan‐Meier estimates of survival for patients in different groups. *p < 0.05. D) Representative mIHC images of different stage tumor tissue showing co‐localization of CX3CL1 and Treg cells. Green, CX3CL1; Red, CD4; Orange, CD25; Purple, PANCK; Blue, DAPI. Scale bars, 200 µm. E) Statistics analysis of the ratio of CD4^+^CD25^+^/CD4^+^ in the adjacent normal, low‐grade tumor, and high‐grade tumor tissues in mIHC‐stained tissue microarrays. One‐way ANOVA test of variance, ns, non‐significant, *p < 0.05. F) Linear matching of the correlation between CX3CL1^+^/PANCK^+^ ratio and CD4^+^CD25^+^ /CD4^+^ ratio. G) Tumor tissues in (Figure S7A, Supporting Information) were digested into single cells, and flow cytometry analysis of the ratio of CD25^+^CD127^−^ (Treg) cells in CD4^+^ T cells. *n* = 5, one‐way ANOVA test of variance, ns, non‐significant, *p < 0.05, ***p < 0.001. H) Tumor tissues in (Figure S7B, Supporting Information) were digested into single cells, and flow cytometry analysis of the ratio of CD25^+^CD127^−^ (Treg) cells in CD4^+^ T cells. *n* = 6, one‐way ANOVA test of variance, ns, non‐significant, **p < 0.01, ***p < 0.001.

To explore the potential of CX3CL1 as a prognostic indicator in clinical samples and validate its correlation with Treg infiltration, we conducted multiplex immunofluorescence (mIHC) staining of a tissue microarray containing 100 CRC tissue samples with antibodies against CX3CL1, CD4, CD25, and PANCK (a tumor cell marker). Given that CD25^+^ Tregs are an independent risk factor for a poor prognosis in patients with CRC, we used CD4 and CD25 as Treg markers instead of CD4 and FOXP3.^[^ ^18^ ^]^ By the 7th edition of the American Joint Committee on Cancer (AJCC) cancer staging system, we categorized tumor tissues with a grade <T3 as low‐grade tumors and those with a grade ≥T3 as high‐grade tumors. We observed a lower CX3CL1^+^/PANCK^+^ ratio in adjacent noncancerous tissues and low‐grade tumor tissues, while the CX3CL1^+^ tumor cell ratio was significantly greater in high‐grade tumor tissues (**Figure** **6**A,B). We divided the samples into a high‐expression group (CX3CL1^High^) and a low‐expression group (CX3CL1^Low^). K‒M survival analysis revealed a significantly poorer prognosis in the high‐expression group than in the low‐expression group (Figure 6C).

Additionally, we observed significant colocalization of CX3CL1 and CD4^+^CD25^+^ Treg cells in the PANCK^−^ stromal region (Figure 6D). Upon statistical analysis, we found an increase in Treg infiltration in high‐grade tumors and a positive correlation between the fluorescence intensity of CX3CL1 and the extent of Treg infiltration (Figure 6E,F). Therefore, the above results demonstrated that CX3CL1 served as a prognostic indicator in CRC and promoted the infiltration of Treg cells in the tumor microenvironment.

Next, we validated the clinical relevance of CX3CL1 as a therapeutic target through in vivo experiments in mice. We divided humanized CRC subcutaneous mice (as described in Figure 4I) into four groups. One week after tumor transplantation, we initiated drug treatments and measured tumor size using calipers every three days. One group served as the control, while the remaining three groups received different treatments: one group received the CX3CR1 inhibitor (JMS‐17‐2, 10 mg kg^−1^) once daily, another group received the PD1 antibody (250 µg per mouse) every three days, and the third group received a combination of both drugs. When the living tumor burden reached 2000 mm^3^ in any mouse, we euthanized all the mice and extracted the tumors. The experimental results showed that the CX3CR1 inhibitor significantly suppressed tumor growth and achieved treatment effects similar to the PD‐1 antibody. Furthermore, the CX3CR1 inhibitor combined with the PD‐1 antibody exhibited enhanced effects, resulting in the best antitumor efficacy (Figure S7A,B, Supporting Information). We digested the tumor tissues into single cells for flow cytometry analysis. We found that only the group receiving the CX3CR1 inhibitor exhibited a significant reduction in Treg cells. In contrast, the PD‐1 antibody group did not show a significant change in the proportion of Treg cells from that in the control group (Figure 6G,H). These data further confirmed the critical role of CX3CL1‐CX3CR1 signaling in promoting the infiltration and function of Treg cells.

## 3 Conclusion

Tumor cells continuously undergo metabolic reprogramming to adapt to the microenvironment's extreme and unstable energy supply conditions, which reshapes the physical and chemical characteristics of the microenvironment.^[^ ^19^ ^]^ Previous studies have indicated that metabolic reprogramming byproducts, such as lactate and reactive oxygen species (ROS), promote tumor progression by suppressing the function of immune cells.^[^ ^20^, ^21^ ^]^ However, this represents a long‐term and macroscopic impact. From the perspective of dynamic regulation within micro‐communities, tumor cells in extreme conditions need to swiftly adapt to perturbations in metabolism and immune challenges. Our previous research indicates that SIRT1 helps tumor cells cope with glucose‐deficient environments by mediating the glucolipid metabolic reprogramming, however, the impact of this process on anti‐tumor immunity remains unclear.^[^ ^3^ ^]^ Thus, further exploration of whether SIRT1 participates in immune suppression is warranted. This study revealed that SIRT1 promotes the secretion of CX3CL1 in CRC cells by deacetylating FOXO1. CX3CL1‐CX3CR1 signaling plays a significant role in driving modestly functional TCF7^+^ Treg cells to a more potent immunosuppressive TNFRSF9^+^ Treg phenotype. Therefore, we identified SIRT1 as one of the key molecules that contributes to the formation of a pro‐tumorigenic CRC microenvironment through both metabolic and immunological ways.

Transcription factors often drive metabolic reprogramming, and studying the immediate effects of these factors on the paracrine secretion of tumor cells and subsequent immune effects is important for restoring an authentic metabolic‐immune regulatory network. During certain “vulnerable” moments in tumor progression, such as nutrient deprivation, hypoxia, and metastasis, robust metabolic adaptation and a suitable immune environment are crucial for the survival of malignant cells. Emerging research suggests that these stress conditions are constantly changing, but our current understanding of the metabolic‐immune coordination process in the tumor microenvironment is superficial. While tumor cells employ variable mechanisms to carry out metabolic reprogramming, several classical transcription factors, such as members of the STAT family, the Sirtuin family, and the HIF family, have been demonstrated to play crucial roles in metabolic conversion.^[^ ^22^, ^23^, ^24^ ^]^ We believe these transcription factors' effects are not limited to metabolism alone. Since releasing cytokines or chemokines is one of the most direct and efficient ways for tumor cells to influence the immune system, we explored the specific mechanisms by which metabolic reprogramming of CRC cells affects the immune environment through a transcription factor‐chemokine model.

With the increased usage of high‐throughput sequencing technologies, researchers have begun to re‐examine immune cells traditionally considered to exhibit definite phenotypes. A recent study indicated that exhausted CD8^+^ T cells exhibit intrinsic heterogeneity. Through transcription factor analysis via a single‐cell CRISPR screening system, the RBPJ‐IRF1 axis was shown to drive terminal differentiation toward exhausted cell phenotypes.^[^ ^25^ ^]^ However, major knowledge gaps remain regarding the gene regulatory networks and corresponding differentiation directions that govern other immunosuppressive cells, such as Tregs, tumor‐associated macrophages, and MDSCs. Pancancer analysis revealed that TNFRSF9^+^ Tregs exert potent immunosuppressive effects in the tumor microenvironment across multiple cancer types, but there are still questions regarding their origin and regulatory factors. Chemokines are considered to play a crucial role in differentiating CD4^+^ T cells into Tregs and the functional differentiation of Tregs.^[^ ^26^ ^]^ Through the analysis of single‐cell RNA sequencing data for T cells from CRC patients in public databases, we determined that the CX3CL1‐CX3CR1 signaling pathway may be involved in inducing the TNFRSF9^+^ phenotype in Treg cells. These findings were further validated via analysis of our sequencing data, as well as through in vivo and in vitro experiments. Moreover, we found that CX3CL1‐CX3CR1 exert its functional effects by activating the transcription of BTG2 and SATB1. In a healthy state, BTG2 is associated with maintaining a quiescent state of T cells, aligning with the functional characteristics of Tregs.^[^ ^27^ ^]^ Moreover, SATB1 has been demonstrated to be closely associated with the development of Tregs.^[^ ^28^ ^]^ In conclusion, our study reveals a potential mechanism wherein metabolic reprogramming of tumor cells drives the functional differentiation of Tregs.

## 4 Experimental Section

### Cell Culture

HCT116 and SW480 cell lines were purchased from ATCC, and the MC38 cell line was obtained from NCI/NIH. All cell lines were propagated and passaged as adherent cell cultures. MC38 cells were maintained in a complete DMEM (Gibco) medium containing 10% fetal bovine serum (FBS) (Gibco) and 100 U ml^−1^ penicillin/streptomycin. HCT116 cells were maintained in complete McCOY's 5A (Gibco) medium containing 10% fetal bovine serum (FBS) and 100 U ml^−1^ penicillin/streptomycin (Gibco). SW480 cells were maintained in a complete L15 (Gibco) medium containing 10% fetal bovine serum (FBS) and 100 U ml^−1^ penicillin/streptomycin. MC38 and HCT116 cells were maintained in adherent conditions at 37 °C in a humidified atmosphere containing 5% CO_2_. SW480 cells were maintained in adherent conditions in airtight culture bottles at 37 °C. The medium was replaced three times weekly, and the cells were passaged using 0.05% trypsin/EDTA (Gibco) and preserved at early passages.

### Transcriptomic Analysis with the Cancer Genome Atlas (TCGA) Datasets—Data Preparing

The transcriptomic and clinical data for colorectal cancer (COAD) were retrieved using the GDC download Tool from The Cancer Genome Atlas (TCGA) database (https://portal.gdc.cancer.gov). All data were normalized by log2 transformation.

### Transcriptomic Analysis with the Cancer Genome Atlas (TCGA) Datasets—Glucolipid Metabolism Consensus Clustering Analysis

The 126 glucolipid metabolism genes significantly associated with the survival prognosis of the CRC sample in the TCGA database were obtained by univariate Cox regression analysis. The mRNA expression matrix was used as the input of consensus clustering analysis to identify the glucolipid metabolic phenotype. Consensus clustering was performed using the ConsensusClusterPlus R package (v.1.58.0). Consequently, the consensus matrix at k = 4 demonstrated distinct delineation between clusters. The empirical cumulative distribution function (CDF) plot initially revealed pronounced optimal separation. Selecting k = 4 resulted in a minimal proportion of ambiguous clustering (PAC) and notably correlated with patient survival outcomes.

### Transcriptomic Analysis with The Cancer Genome Atlas (TCGA) Datasets—Evaluation of the Correlation between SIRT1 and Immune Cell Subtype Infiltration

The CIBERSORT algorithm (https://github.com/jason‐weirather/CIBERSORT) assessed immune cell infiltration in CRC samples from the TCGA database. LM22 was employed as a reference expression profile. The LM22 signature matrix defined 22 infiltrating immune cell components. Cell types were selected that were more abundant in the tumor microenvironment and were potentially involved in immune suppression, including macrophage subtypes (M0 macrophages, M1 macrophages, and M2 macrophages) and T cells (CD8^+^ T cells, naïve CD4^+^ T cells, memory CD4^+^ T cells, Tfh cells, regulatory T cells, and γδ T cells). *P*‐values and root mean square errors were determined for each expression file in CIBERSORT. Only data with CIBERSORT *p*‐values < 0.05 were retained for subsequent analysis. The output was directly integrated to generate a comprehensive matrix of immune cell scores. After integrating this matrix with the gene expression matrix, Spearman correlation coefficients between SIRT1 and immune cells were calculated and visualized.

### Transcriptomic Analysis with The Cancer Genome Atlas (TCGA) Datasets—WGCNA Based on Cancer–Immunity Cycle Scores

A weighted gene co‐expression network was constructed utilizing the “WGCNA” package in R software. Standardized transcriptome expression data from colorectal adenocarcinoma (COAD) was employed as the input matrix for the WGCNA process. Immune cycle scores for COAD specimens, derived from the TIP database (http://biocc.hrbmu.edu.cn/TIP/), were integrated as clinical phenotypes for the WGCNA. The expression matrix was converted into an adjacency matrix and subsequently transformed into a topological overlap matrix. Gene clustering was conducted based on topological overlap using the average‐linkage hierarchical clustering approach. Adhering to the hybrid dynamic tree‐cutting criteria, a minimum threshold of 30 genes was established for each network module. Key genes within each module were pinpointed, and the modules were subjected to clustering with a height cutoff threshold of 0.25; the grey module was designated for genes that did not neatly fit into any other category. Employing the key genes from each module, the correlation between module membership and clinical phenotypes was calculated, culminating in creating a module‐phenotype correlation heatmap.

### Multiplex Immunohistochemical (mIHC) Staining and Analysis

The tissue microarray was stained with a PANO 7‐plex IHC kit, cat 0004100100 (Panovue, Beijing, China), followed the standard protocol. The slides were deparaffinized in xylene, rehydrated, and washed in tap water before boiling in antigen retrieval. Use tissue markers to draw tissue areas and Antibody Diluent / Block for protein blocking (CST). All antigens were labeled separately, including primary antibody incubation (CX3CL1: Abcam, CD25: Abcam, CD4: ZSGB‐BIO, PNCK: MERCK), secondary antibody incubation, and TSA visualization, followed by labeling the next antibody. Primary antibodies were incubated for 1 h at room temperature. Next, incubation with Opal Polymer HRP was performed at 37 °C for 10 min. TSA visualization was performed with the PANO 7‐plex IHC kit. Microwave treatment was performed to remove the Ab TSA complex with antigen retrieval. The slide was finished with DAPI for 5 min and was enclosed in an antifade mounting medium. Slides were scanned using the Olympus VS200 MTL (Olympus Germany). Image analysis software Qupath was used to build the algorithm by checking, training, and confirming the steps. TUMOR, STROMA, and other tissue classifications were constructed in the algorithm construction, and cases were recorded. Accurately recognize and count cells using Qupath software and set reasonable thresholds to identify positive cells. Counting the number of all cells and the number of positive cells.

### Single‐Cell RNA Sequencing and Analysis—RT & Amplification and Library Construction

Surgically resected tumor samples were dissociated into single cells, and CD45^+^ cells were isolated using flow cytometry for single‐cell RNA sequencing. Single‐cell suspensions, prepared at a concentration of 2 × 10^5^ cells ml^−1^ in PBS (HyClone), were loaded onto a microwell chip using the Singleton Matrix Single Cell Processing System. Barcoding Beads were then harvested from the chip, allowing for the reverse transcription of mRNA captured by these beads to synthesize cDNA, followed by PCR amplification. The resulting amplified cDNA was fragmented and subsequently ligated with sequencing adapters. Libraries for scRNA‐seq were prepared per the GEXSCOPE Single Cell RNA Library Kits (Singleton) protocol. Individual libraries, normalized to a 4 nM concentration, were pooled and subjected to sequencing on an Illumina Nova Seq 6000 platform, employing 150 bp paired‐end reads.

### Single‐Cell RNA Sequencing and Analysis—Quality Control, Dimension Reduction, and Clustering

Cells were filtered by gene counts of more than 200 or less than 6000 and mitochondrial content of no more than 20%. Post‐filtering, 33130 cells met the criteria and were carried forward for subsequent analyses. Tools were employed from Seurat (v.4.2.0) to facilitate dimensionality reduction and clustering. The NormalizeData and ScaleData functions were utilized to standardize gene expression levels across all cells, followed by identifying the top 2000 highly variable genes using the FindVariableFeatures function, which was then used for principal component analysis (PCA). The batch effect between samples was removed by Harmony (v.0.1.0). Finally, the UMAP algorithm was applied to visualize cells in a 2D space.

### Single‐Cell RNA Sequencing and Analysis—RNA velocity

For RNA velocity, the BAM file and reference genome GRCh38 (hg38) were used in the analysis with velocity (v.0.6) and scVelo (v.0.3.0) in Python. The result was projected to the UMAP plot from the Seurat clustering analysis for visualization consistency.

### GSEA Analysis

Gene Set Enrichment Analysis was executed via R‐based clusterProfiler (v.4.2.2) on normalized expression data. GSEA ranked genes by phenotype correlation, calculating Enrichment Scores (ES) and Normalized Enrichment Scores (NES) through 1000 permutations. Significance was determined by a False Discovery Rate (FDR) < 0.25, with significant gene sets visualized using enrichplot.

### T Cell Isolation and Cultivation

The Transfusion Department at Southwest Hospital provided human peripheral blood mononuclear cells (PBMCs). PBMCs were diluted with the same volume of PBS (Solarbio), and then lymphocytes were separated by gradient centrifugation with Lymphocyte Separation Medium (Human) (Solarbio). After resuspending the lymphocytes with a complete RPMI‐1640 medium (Gibco), cells were placed in a culture dish and maintained overnight. Collecting supernatant containing T cells and discarding adherent cells. The sorted cells were stimulated with anti‐CD3 (2 µg ml^−1^) (Biolegend), anti‐CD28 (2.5 µg ml^−1^) (Biolegend) antibodies and IL‐2 (1000 U ml^−1^) (Biolegend) for 48 h and cultured in RPMI‐1640 (10% FBS).

### In Vivo Experiments—Humanized Xenograft Mice

Six‐week‐old male NOD/ShiLtJGptPrkdc^em26Cd52^Il2rg^em26Cd22^/Gpt (NCG) mice were purchased from the GemPharmatech Co., Ltd (Jiangsu, China) and acclimated for one week. Each mouse was injected subcutaneously in the right groin with 1 × 10^6^ HCT116 cells, and then the mice were divided into two groups randomly. After one week, 1 × 10^7^/200 ul CD3/CD28 antibody‐activated human T cells were injected into the tail vein of each mouse every week. The PD‐1 treated group was receiving intraperitoneal injections of anti‐PD‐1 (BioXcell) 200 ug per mouse every three days. The JMS‐17‐2 treated group received intraperitoneal injections of the CX3CR1 inhibitor JMS‐17‐2 (MCE) 10 mg k^−1^g daily. The Quetmolimab treated group received intraperitoneal injections of the CX3CL1 neutralizing antibody Quetmolimab (Selleck) 200 µg per mouse every 3 days. The control group received intraperitoneal injections of the same volume solvent. Tumor sizes were measured every three days in two dimensions with the caliper and calculated using the formula (L × W^2^)/2, where L is the length, and W is the width. When the living tumor volume in any subject mouse reached 2000 mm^3^, this was designated the experimental endpoint. Subsequently, all mice were humanely euthanized, and tumor specimens were harvested for subsequent flow cytometry analysis.

### In Vivo Experiments—Allograft Mice

Six‐week‐old male C57BL/6 mice were purchased from the Hubei Benente Biotechnology Co., Ltd (Hubei, China) and acclimated for one week. Each mouse was injected subcutaneously in the right groin with 1 × 10^6^ MC38 or 1 × 10^6^ Sirt1‐KO MC38 cells. The experimental and control groups were injected subcutaneously in the right groin with 1 × 10^6^ Sirt1‐KO MC38 and WT‐MC38 cells, respectively; no additional treatment was applied. Tumor sizes were measured every three days in two dimensions with the caliper and calculated using the formula (L×W^2^)/2, where L is the length, and W is the width. When the living tumor volume in any subject mouse reached 2000 mm^3^, this was designated the experimental endpoint. Subsequently, all mice were humanely euthanized, and tumor specimens were harvested for subsequent flow cytometry analysis.

### Primary Cell Isolation and Flow Cytometry Analysis (FACS)

After the mice were sacrificed, the separation subcutaneous transplant tumors were cut into ≈1 × 1 mm^3^ pieces with scissors. Digesting tumors in a shaker for 1 h with digest diluent, RPMI‐1640 containing 1 g L^−1^ collagenase I (BioFroxx), 1 g L^−1^ collagenase IV (BioFroxx), 10 mg L^−1^ DNase I (BioFroxx) at 37 °C. Collecting single‐cell suspension after filtering with 70 um filter. Cells were stained with fluorescent antibodies and analyzed by flow cytometry. Cells were first stained with Zombie NIR Fixable Viability Kit (BioLegend) following the manufacturer's instructions for live/dead criteria. Subsequent surface markers staining was performed in FACS buffer containing 1×PBS with 2% FBS. Intracellular staining for nuclear proteins was performed using the eBioscience FoxP3/Transcription Factor Staining Buffer Set (ThermoFisher Scientific), and cytoplasmic proteins, such as cytokines, were stained by the Fixation/Permeabilization Solution Kit (BD Biosciences). Intracellular cytokine staining was performed after a 4 h stimulation with Cell Activation Cocktail (with Brefeldin A) (Biolegnd) at 37 °C.

### Western Blotting

Cells were lysed with an ice‐cold protein lysis solution containing phenylmethanesulfonyl fluoride (PMSF) (Beyotime). Total protein concentration was measured by the BCA Protein Assay Kit (Beyotime) according to the manufacturer's instructions. Protein was loaded 10–30 ug equally for separating by SDS‐PAGE and transferred to nitrocellulose membranes (0.45 µm). After blocking with TBST containing 5% BSA, membranes were incubated with primary and secondary antibodies. Blots were visualized with Affinity ECL Reagent (femtogram) (Affinity). The grayscale values of western blotting bands were analyzed using ImageJ.

### Co‐Immunoprecipitation

Proteins interacting with A/G Dynabeads were pulled down for Western blotting analysis using Pierce Classic Magnetic IP/Co‐IP Kit (Thermo Fisher). Cells were lysed with an ice‐cold protein lysis solution containing phenylmethanesulfonyl fluoride (PMSF). The protein supernatant was incubated with a specific antibody overnight at 4 °C for target protein immunoprecipitation. Then, the supernatant was incubated with dynasts for 2 h at 4 °C. Sorting bead‐coupled proteins by magnetic frame and eluting protein for Western blotting analysis.

### Confocal Microscopy

Tumor cells were seeded on the confocal dish. Cells were fixed using 4% PFA (Biosharp) and permeabilized using 0.3% Triton (Biosharp). After permeabilization, cells were blocked with 1% BSA (BioFroxx) in PBS for 30 min and incubated with primary antibody overnight at 4 °C. Then, probed with Alexa 488‐conjugated rabbit anti‐mouse (CST) or Alexa 555‐conjugated mouse anti‐rabbit (CST) secondary. Finally, staining nuclear using DAPI (CST). An LSM900 fluorescence confocal microscope (Zeiss) under a 40x lens was used to obtain confocal fluorescence images.

### Statistical and Quantitative Analysis of Staining and Microscopy Images

For the statistical analysis of the confocal fluorescence images, the relevant information was first quantified using ImageJ software and then performed graphical and statistical analysis using PRISM. Differences were tested by using a one‐way ANOVA followed by a Tukey's multiple comparisons test (*p < 0.05; **p < 0.01, ***p < 0.001). Average values were indicated in the plots.

### Other Statistical Analysis

The data statistic was performed using PRISM. Paired or unpaired Student's *t*‐test (two tails) was used to compare experiments with only two groups. One‐way ANOVA with multiple comparisons was performed for experiments with more than two groups. Kaplan‐Meier assay and Log‐rank test were used for survival analysis (ns: no significance; *p < 0.05; **p < 0.01, ***p < 0.001).

### Ethics Approval and Consent to Participate

All the animal experiments were performed in accordance with institutional guidelines and received ethical approval from the Animal Ethics Committee of Third Military Medical University. Human tumor tissue samples were approved by the Institutional Review Board of Southwest Hospital (#KY2023115). Patients were informed of the trial content and given consent.

## Conflict of Interest

The authors declare no conflict of interest.

## Author Contributions

R.Z., X.Z., and L.L. contributed equally to this work. Z.W. and Y.D. worked on conceptualization. R.Z., Z.W., and Y.D. worked on data curation. Z.W., R.Z., X.Z., and L.L. worked on formal analysis. Z.W., Y.D., J.L., and H.L. worked on funding acquisition. R.Z., X.Z, Y.W., Z.B., H.J., T.L., Y.S., X.W., F.L., C.Z., F.Z., and Q.Q. worked on investigation. Z.W., R.Z., X.Z., Y.W., and H.J. worked on methodology. Z.W. and Y.D. worked on project administration. X.Z., Z.W., and Y.D. worked on resources. Z.W., R.Z., and H.P. worked on software. Y.D., Z.W., J.L., and H.L. worked on supervision. R.Z., X.Z., Y.W., Z.W., and Y.D. worked on validation. Z.W., R.Z., X.Z., L.L., and H.P. worked on visualization. Z.W., R.Z., X.Z., L.L, H.L., J.L., and Y.D. worked on wrote the original draft. Z.W., R.Z., X.Z., L.L, H.L., J.L., and Y.D. worked on wrote, reviewed, and edited the draft.

## Supporting information



Supporting Information

## Data Availability

The raw sequence data reported in this paper have been deposited in the Genome Sequence Archive (Genomics, Proteomics & Bioinformatics 2021) at the National Genomics Data Center (Nucleic Acids Res 2022), China National Center for Bioinformation / Beijing Institute of Genomics, Chinese Academy of Sciences (GSA‐Human: HRA006612 and HRA006575) that are publicly accessible at https://ngdc.cncb.ac.cn/gsa‐human. This paper has also analyzed existing, publicly available data. These accession numbers or relevant publications/database sources for the datasets are listed in the text. Other data that support the findings of this study are available from the corresponding author upon reasonable request.
