# Supplementary material for: Metabolic‐Immune Suppression Mediated by the SIRT1‐CX3CL1 Axis Induces Functional Enhancement of Regulatory T Cells in Colorectal Carcinoma
Source: Adv Sci (Weinh). 2025 Jan 9;12(17):2404734. doi: 10.1002/advs.202404734 (PMC12061293; doi:10.1002/advs.202404734)
Supplement: Supplementary file 1 — Supporting Information [file ADVS-12-2404734-s001.docx]

**Metabolic-immune suppression mediated by the SIRT1-CX3CL1 axis induces functional enhancement of regulatory T cells in colorectal carcinoma**

Ruiyang Zi^1,#^, Xiang Zhao^1,#^, Limei Liu^3,#^, Yijie Wang^1^, Zhiheng Bian^1^, Haoran Jiang^1^, Taorui Liu^1^, Yixin Sun^1^, Han Peng^1^, Xuesong Wang^1^, Fanghao Lu^1^, Chao Zhang^1^, Fan Zhang^1^, Qing Tan^1^, Houjie Liang^1,^*, Jianjun Li^1,^*, Zhihao Wei^1,2,^* and Yan Dong^1,^*

Correspondence to: yandong@tmmu.edu.cn, weizhihao7768@tmmu.edu.cn, jianjunli@tmmu.edu.cn, lianghoujie@sina.com

**This file includes:**

Figs. S1 to S7

**Supplementary Figures**

**
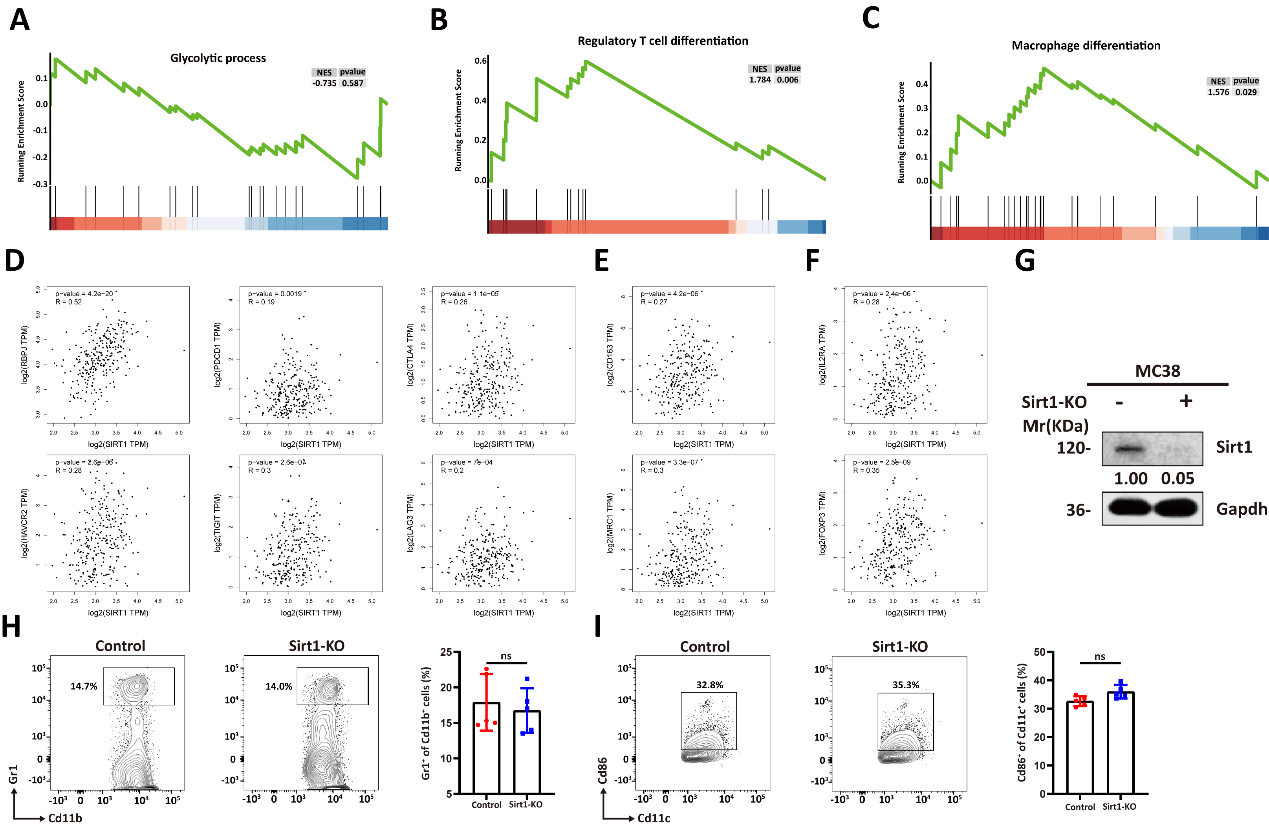
**

**Figure S1. SIRT1 is closely related to the immunosuppressive tumor microenvironment.**

(A) Enrichment score of the ‘Glycolytic process’ in C4 patients versus C1 patients, analyzed by GSEA using RNA-seq data of TCGA-COAD cohort.

(B) Enrichment score of the ‘Regulatory T cell differentiation’ in C4 patients versus C1 patients, analyzed by GSEA using RNA-seq data of TCGA-COAD cohort.

(C) Enrichment score of the ‘Macrophage differentiation’ in C4 patients versus C1 patients, analyzed by GSEA using RNA-seq data of TCGA-COAD cohort.

(D) GEPIA analysis of the correlation between SIRT1 and T cell exhaustion-related genes.

(E) GEPIA analysis of the correlation between SIRT1 and marker genes of M2-macrophages.

(F) GEPIA analysis of the correlation between SIRT1 and marker genes of Treg.

(G) Sirt1 knockout efficiency of MC38 was assessed using immunoblotting.

(H) Flow cytometry analysis of the ratio of Gr1^+^ cells in Cd11b^+^ cells. n=5, student *t*-test of variance, ns, non-significant.

(I) Flow cytometry analysis of the ratio of Cd86^+^ cells in Cd11c^+^ T cells. n=5, student *t*-test of variance, ns, non-significant.


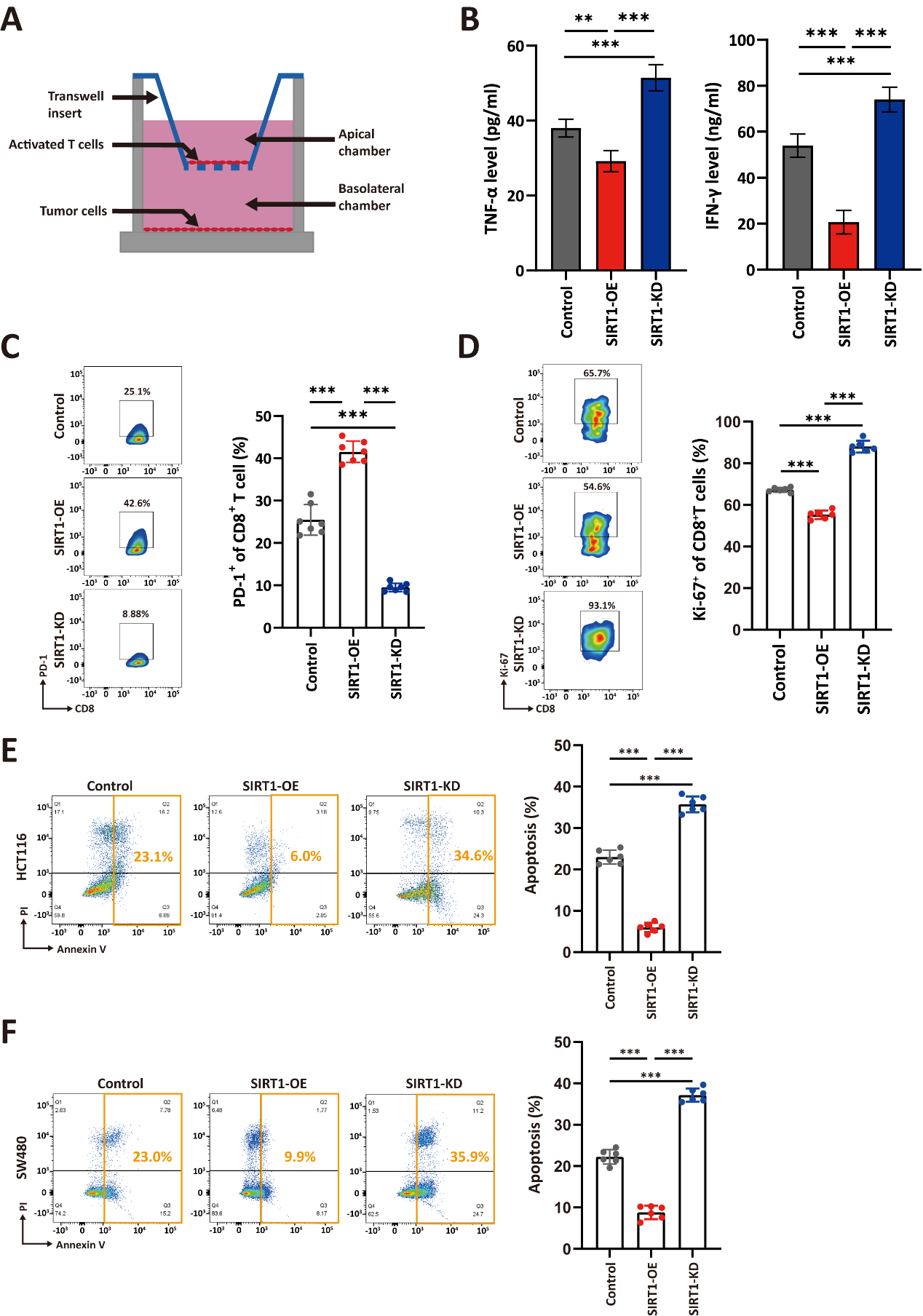


**Figure S2. SIRT1 promotes the expression of CX3CL1 in colorectal tumor cells**

(A) Co-culture of activated T cells and tumor cells, activated T cells placed in the apical chamber and tumor cells placed in the basolateral chamber for cultivation.

(B) ELISA of effector cytokines TNFα (left), IFNγ (right) in the supernatant of activated T cells co-culture with indicated SW480 cells. n=6, one-way ANOVA test of variance, ***p* < 0.01, ****p* < 0.001.

(C) Activated T cells were co-cultured with indicated SW480 cells. Flow cytometry analysis of the ratio of PD-1^+^ cells in CD8^+^ T cells. n=7, one-way ANOVA test of variance, ****p* < 0.001.

(D) Activated T cells were co-cultured with indicated SW480 cells. Flow cytometry analysis of the ratio of Ki67^+^ cells in CD8^+^ T cells. n=6, one-way ANOVA test of variance, ****p* < 0.001.

(E-F) Applying illustrated treatments to HCT116 and SW480 cells, co-culturing them with activated T cells for 72 hours. Flow cytometry assessed the apoptotic rates of the tumor cells in each experimental group. n=6, one-way ANOVA test of variance, ****p* < 0.001.

**
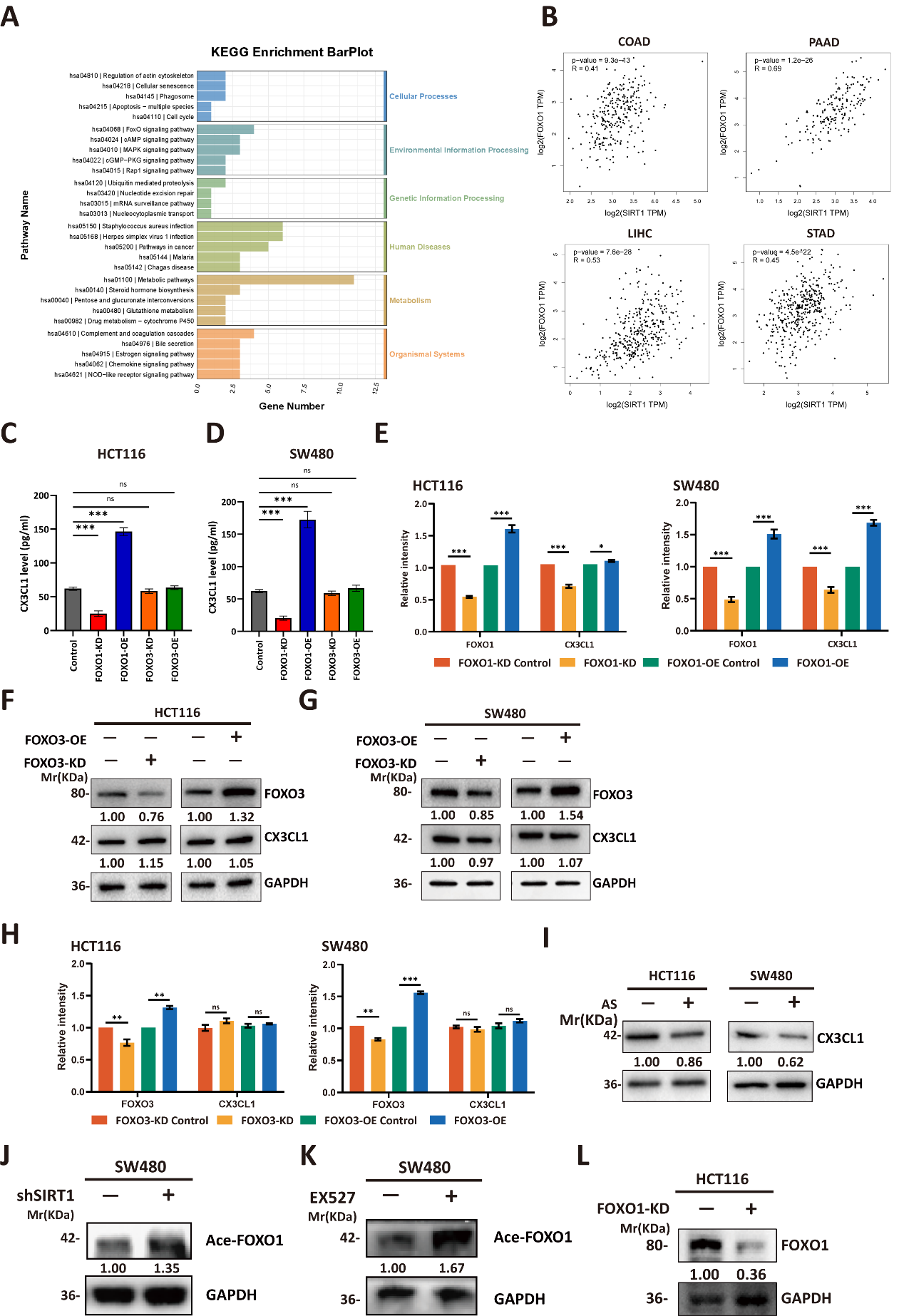
**

**Figure S3. SIRT1 activates FOXO1 to promote CX3CL1 expression**

(A) In the SIRT1-OE HCT116 cells relative to the SIRT1-WT, the upregulated DEGs were subjected to KEGG pathway enrichment analysis. The x-axis represents the number of DEGs included in the pathway, the y-axis represents the pathway names, and the color indicates the first-level classification of KEGG.

(B) GEPIA analysis of the correlation between SIRT1 and FOXO1 in different gastrointestinal cancers.

(C-D) ELISA of CX3CL1 in supernatant of FOXO1-KD, FOXO1-OE, FOXO3-KD, FOXO3-OE and control HCT116 cells (C) or SW480 cells (D). n=3, one-way ANOVA test of variance, ns, non-significant, ****p* < 0.001.

(E) Analyze the grayscale values of the western blot bands in Figure 3D and 3E using ImageJ and perform statistical analysis on the results of three technical replicates. n=3, one-way ANOVA test of variance, **p* <0.05, ****p* < 0.001.

(F-G) FOXO3 and CX3CL1 levels of FOXO3-KD, FOXO3-OE, and control HCT116 (D) or SW480 (E) were assessed using immunoblotting.

(H) Analyze the grayscale values of the western blot bands in Supplementary Figure 3F and 3G using ImageJ and perform statistical analysis on the results of three technical replicates. n=3, one-way ANOVA test of variance, ns, non-significant, ***p* < 0.01, ****p* < 0.001.

(I) After being treated with FOXO1 inhibitor AS1842856 (1μM, 48h), the CX3CL1 expression level of HCT116 and SW480 cells was assessed using immunoblotting.

(J-K) Acetyl level of FOXO1 in SIRT1-KD (J) or SIRT1 inhibitor EX527 treated SW480 cells (K) was assessed using immunoblotting.

(L) Verification of the knockdown efficiency of FOXO1 in HCT116 cells using immunoblotting.

**
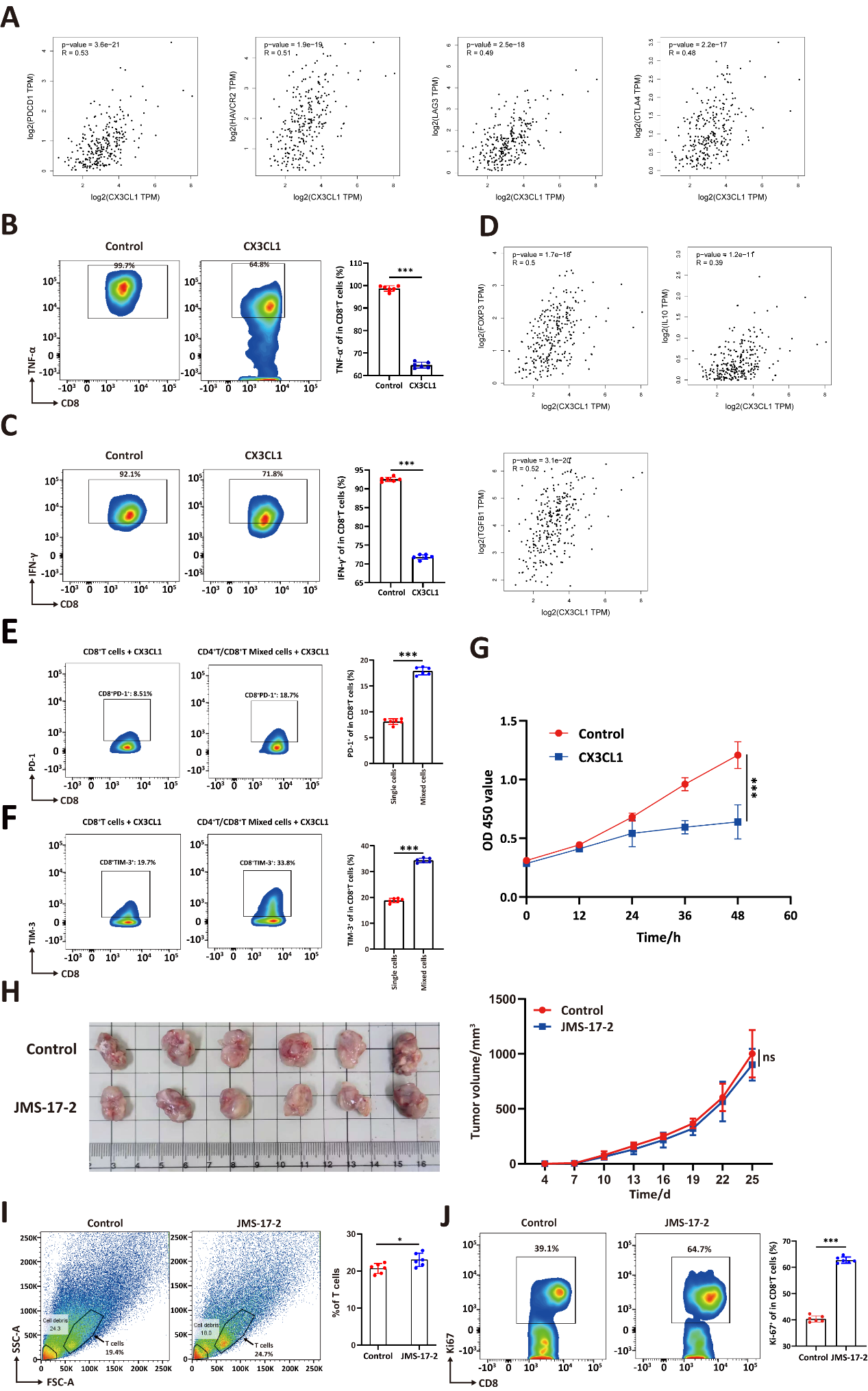
**

**Figure S4. CX3CL1 promotes immune suppression by mediating the function of Treg cells.**

(A) GEPIA analysis of the correlation between CX3CL1 and T cell exhaustion-related genes.

(B-C) Activated T cells co-cultured with HCT116 cells received indicated treatment, and flow cytometry analysis of the ratio of TNFα^+^ cells in CD8^+^ T cells (B); the ratio of IFNγ^+^ cells in CD8^+^ T cells (C). n=6, student *t*-test of variance, ****p* < 0.001.

Flow cytometric analysis effector cytokine of CX3CL1 treated or control activated T cells. Representative flow cytometric profiles and statistical analyses of CD8^+^ double-positive cells (B) andCD8^+^ double-positive cells (C) are shown. n=6, student *t*-test of variance, ****p* < 0.001.

(D) GEPIA analysis of the correlation between CX3CL1 and Treg-related genes.

(E-F) CX3CL1 was added into individual CD8^+^ T cells, or CD4^+^/CD8^+^ T mixed cells, and flow cytometry analysis of the ratio of PD-1^+^ cells (E) and TIM-3^+^ cells (F) in CD8^+^ T cells. n=6, student *t*-test of variance, ****p* < 0.001.

(G) CX3CL1 was added into individual CD8^+^ T cells, or CD4^+^/CD8^+^ T mixed cells. Using CCK8 assay to evaluate the proliferative capacity of CD8^+^ T cells from different groups. n=6, student *t*-test of variance, ****p* < 0.001.

(H) NCG mice with subcutaneous xenografts (10^6^ HCT116) have tail vein injected only CD8^+^ T cells. All mice were divided into two groups and received indicated treatment, tumor tissues were extracted at the experimental endpoint (left). The tumor size was measured every three days using calipers to plot the tumor growth curve (right). Statistical significance was assessed by student *t*-test of variance. ns, non-significant.

(I-J) Tumor tissues in Figure 4J were digested into single cells and flow cytometry analysis of the ratio of T cells in total cells (I) and the ratio of Ki67^+^ cells in CD8^+^ T cells (J). n=6, student *t*-test of variance, **p* < 0.05,****p* < 0.001.

**
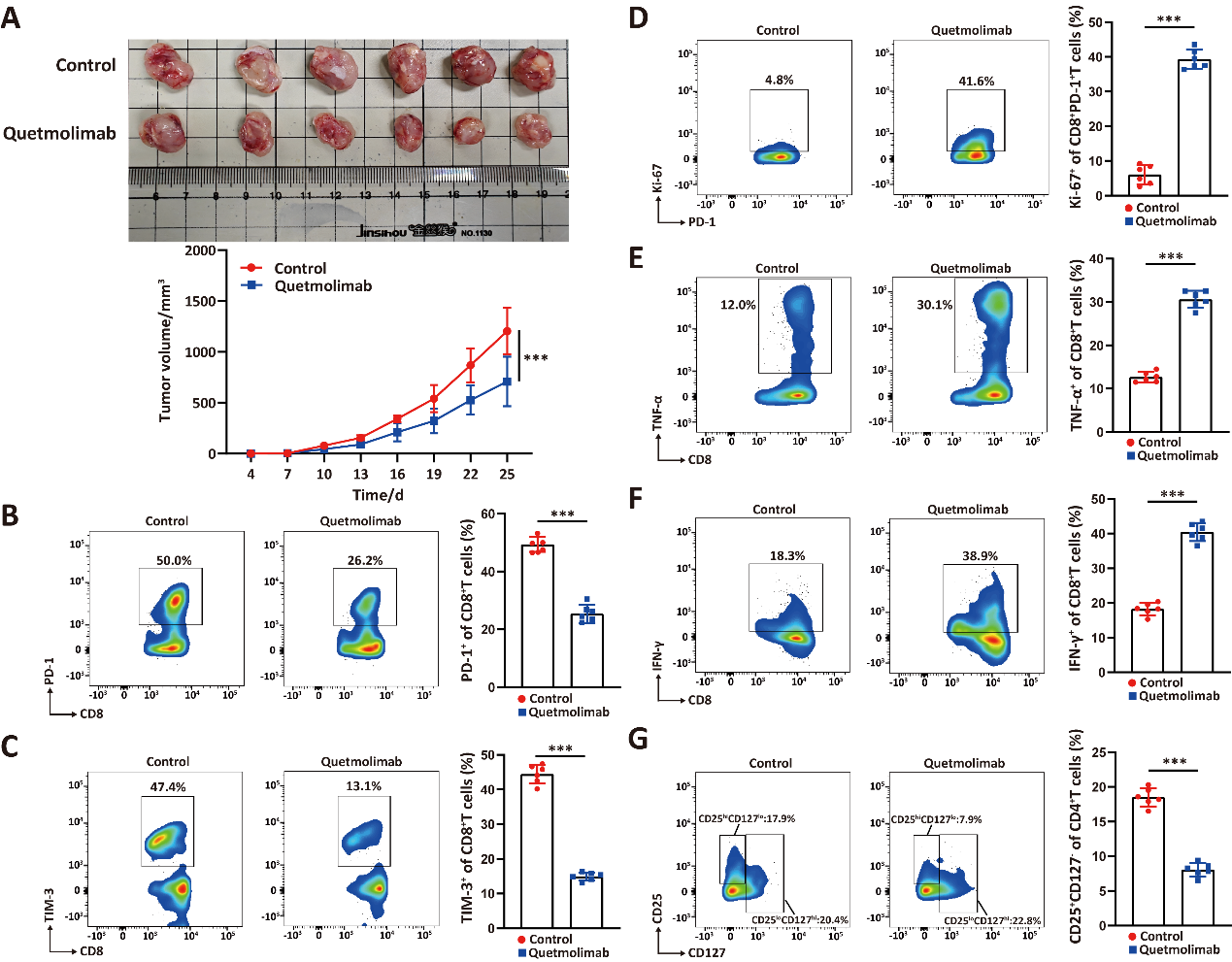
**

**Figure S5. Blocking CX3CL1 enhances the antitumor immune efficacy of T cells.**

(A) Humanized subcutaneous xenograft mice were generated using the method depicted in Figure 4I.NCG mice with subcutaneous xenografts (10^6^ HCT116 cells) have tail vein injected CD4^+^/CD8^+^ T cells. All mice were divided into two groups and received indicated treatment, tumor tissues were extracted at the experimental endpoint (upper panel). The tumor size was measured every three days using calipers to plot the tumor growth curve (bottom panel). Statistical significance was assessed by student *t*-test of variance. ns, non-significant, ****p* < 0.001.

(B-F) Tumor tissues in (A) were digested into single cells, and flow cytometry analysis of the ratio of PD-1^+^ cells in CD8^+^ T cells (B); the ratio of TIM-3^+^ cells in CD8^+^ T cells (C); the ratio of Ki67^+^ cells in CD8^+^ T cells (D); the ratio of TNF-α^+^ and IFN-γ^+^ cells in CD8^+^ T cells (E-F). n=6, student *t*-test of variance, ****p* < 0.001.

(G) Tumor tissues in (A) were digested into single cells, and flow cytometry analysis of the ratio of CD25^+^CD127^-^ (Treg) cells in CD4^+^ T cells. n=6, student *t*-test of variance, ****p* < 0.001.

**
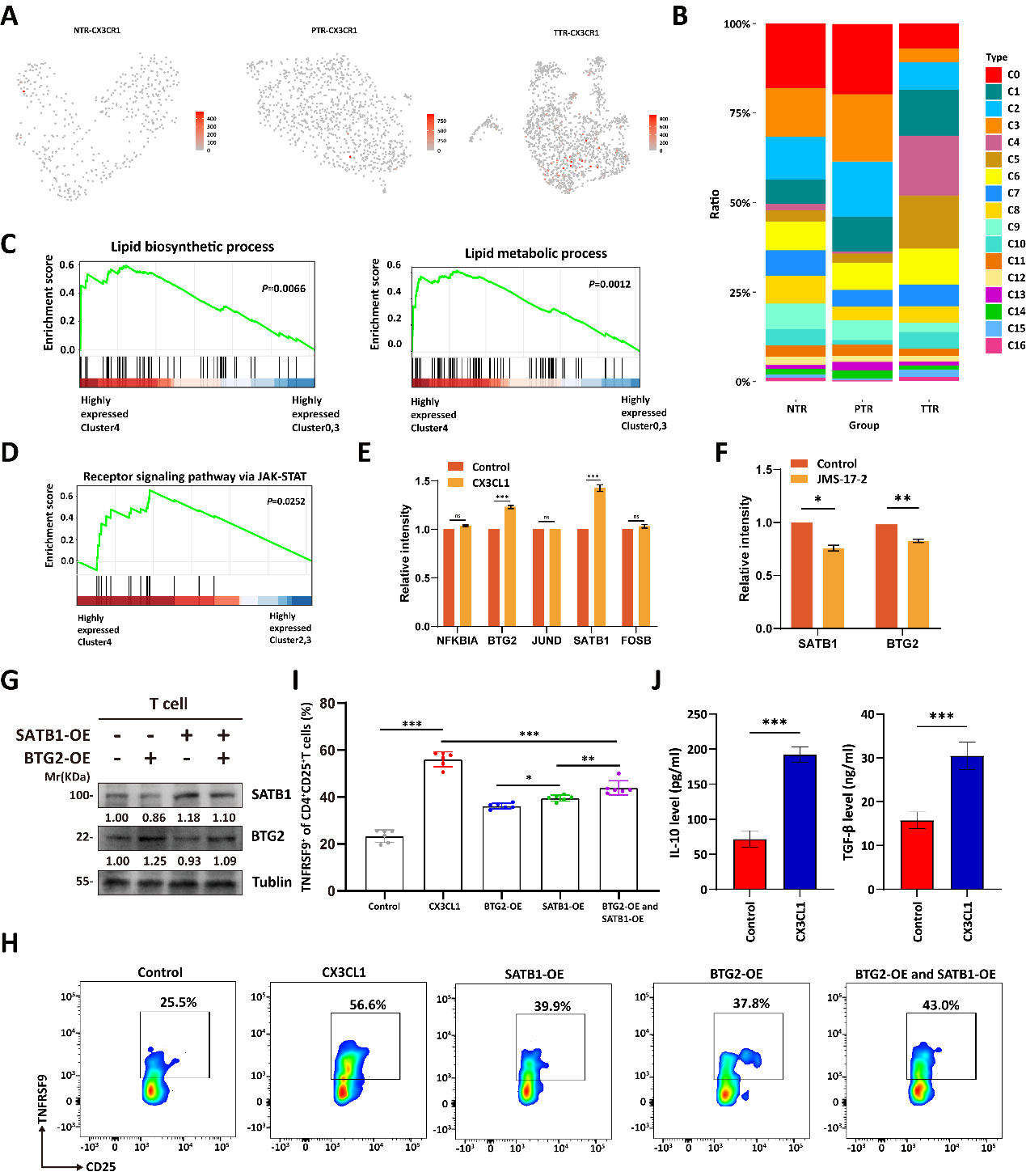
**

**Figure S6. CX3CL1 enhances the function of Treg cells by promoting the TNFRSF9 phenotype.**

(A) Feature plots of CX3CR1 in Treg cells sourced from peripheral blood, adjacent normal tissue, and cancer tissue. NTR, Tregs from adjacent normal colorectal tissues; PTR, Tregs from peripheral blood; TTR, Tregs from CRC.

(B) Stacked bar plot illustrating the composition of Treg subgroups from different samples. NTR, Tregs from adjacent normal colorectal tissues; PTR, Tregs from peripheral blood; TTR, Tregs from CRC.

(C) GSEA analysis of the ‘Lipid biosynthetic process’ and ‘Lipid metabolic process’ enrichment score of Treg in Cluster 1/4 versus Cluster 0/3.

(D) GSEA analysis of the ‘Receptor signaling pathway via JAK-STAT’ enrichment score of Treg in Cluster 1/4 versus Cluster 0/3.

(E-F) Analyze the grayscale values of the western blot bands in Figure 5J and 5K using ImageJ and perform statistical analysis on the results of three technical replicates. n=3, one-way ANOVA test of variance, ns, non-significant, **p* <0.05, ***p* < 0.01, ****p* < 0.001.

(G) Verification of the overexpression efficiency of BTG2 and SATB1 in activated T cells using immunoblotting.

(H-I) Co-culturing CD4^+^ T cells from different groups, as illustrated, with SW480 cells. The ratio of TNFRSF9^+^ cells in CD25^+^ T cells was analyzed by flow cytometry. n=6, student *t*-test of variance, **p* < 0.05, ****p* < 0.01, ****p* < 0.001.

(J) Exogenous addition of CX3CL1 significantly increased the IL-10 (left) and TGF-β (right) levels of CD4^+^ T cells co-cultured with SW480. n=6, student *t*-test of variance, ****p* < 0.001.

**
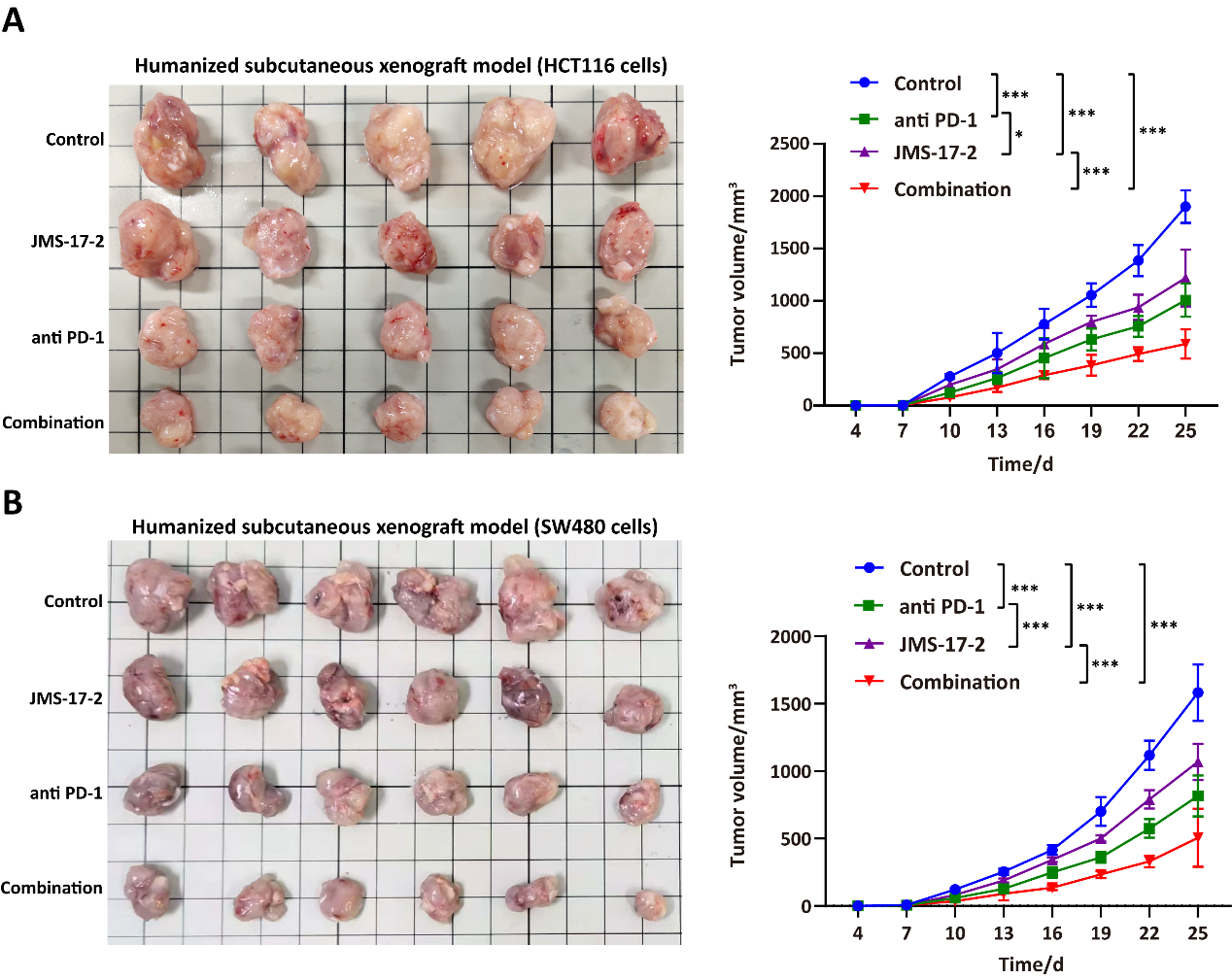
**

**Supplementary Figure 7. Validation of the therapeutic effect of CX3CR1 blockade in humanized CRC subcutaneous mice.**

(A) Representative gross appearance (left) of the subcutaneous xenografts in the indicated groups. Tumor growth curves (right) of xenografts bearing NCG mice inoculated with 10^6^ HCT116 cells. Statistical significance was assessed by two-way ANOVA followed by the Bonferroni post-hoc correction. **p* < 0.05, ****p* < 0.001. The dimensions of each small square are 1 cm×1 cm.

(B) Representative gross appearance (left) of the subcutaneous xenografts in the indicated groups. Tumor growth curves (right) of xenografts bearing NCG mice inoculated with 10^6^ SW480 cells. Statistical significance was assessed by two-way ANOVA followed by the Bonferroni post-hoc correction. ****p* < 0.001. The dimensions of each small square are 1 cm×1 cm.
